# Cerebrovascular amyloid Angiopathy in bioengineered vessels is reduced by high-density lipoprotein particles enriched in Apolipoprotein E

**DOI:** 10.1186/s13024-020-00366-8

**Published:** 2020-03-25

**Authors:** Jerome Robert, Emily B. Button, Emma M. Martin, Luke McAlary, Zoe Gidden, Megan Gilmour, Guilaine Boyce, Tara M. Caffrey, Andrew Agbay, Amanda Clark, Judith M. Silverman, Neil R. Cashman, Cheryl L. Wellington

**Affiliations:** 1 Department of Pathology and Laboratory Medicine, University of British Columbia, Vancouver, British Columbia V6T 1Z3 Canada; 2 Djavad Mowafaghian Centre for Brain Health, University of British Columbia, Vancouver, British Columbia V6T 1Z3 Canada; 3 Present address: Institute of Clinical Chemistry, University Hospital Zurich, 8000 Zurich, Switzerland; 4 Department of Physics and Astronomy, University of British Columbia, Vancouver, British Columbia V6T 1Z1 Canada; 5 Department of Neurology, University of British Columbia, Vancouver, British Columbia V6T 2B5 Canada; 6 School of Biomedical Engineering, University of British Columbia, Vancouver, British Columbia V6T 1Z3 Canada; 7 International Collaboration on Repair Discoveries, University of British Columbia, Vancouver, British Columbia V5Z 1M9 Canada

**Keywords:** Alzheimer’s disease, Cerebrovasculature, High-density lipoprotein (HDL), Cerebral amyloid angiopathy (CAA), Endothelial inflammation, Apolipoprotein (apo)E, Tissue engineering

## Abstract

**Background:**

Several lines of evidence suggest that high-density lipoprotein (HDL) reduces Alzheimer’s disease (AD) risk by decreasing vascular beta-amyloid (Aβ) deposition and inflammation, however, the mechanisms by which HDL improve cerebrovascular functions relevant to AD remain poorly understood.

**Methods:**

Here we use a human bioengineered model of cerebral amyloid angiopathy (CAA) to define several mechanisms by which HDL reduces Aβ deposition within the vasculature and attenuates endothelial inflammation as measured by monocyte binding.

**Results:**

We demonstrate that HDL reduces vascular Aβ accumulation independently of its principal binding protein, scavenger receptor (SR)-BI, in contrast to the SR-BI-dependent mechanism by which HDL prevents Aβ-induced vascular inflammation. We describe multiple novel mechanisms by which HDL acts to reduce CAA, namely: i) altering Aβ binding to collagen-I, ii) forming a complex with Aβ that maintains its solubility, iii) lowering collagen-I protein levels produced by smooth-muscle cells (SMC), and iv) attenuating Aβ uptake into SMC that associates with reduced low density lipoprotein related protein 1 (LRP1) levels. Furthermore, we show that HDL particles enriched in apolipoprotein (apo)E appear to be the major drivers of these effects, providing new insights into the peripheral role of apoE in AD, in particular, the fraction of HDL that contains apoE.

**Conclusion:**

The findings in this study identify new mechanisms by which circulating HDL, particularly HDL particles enriched in apoE, may provide vascular resilience to Aβ and shed new light on a potential role of peripherally-acting apoE in AD.

## Background

As our population ages, the burden of cardiometabolic, neurodegenerative and neuroinflammatory diseases is growing rapidly. Alzheimer’s Disease (AD) is defined by the presence of beta-amyloid (Aβ) deposits and neurofibrillary tangles in the brain and affects over 50 million people worldwide with a global economic burden closed to one trillion US dollars [1]. Despite decades of research, there is not yet an approved disease-modifying therapy for AD, as many drugs that target parenchymal pathologies have been ineffective in attenuating cognitive decline [2]. One exception may be the anti-amyloid immunotherapy aducanumab, which clears amyloid from the brain [3] and appears to slow cognitive decline at the highest dose so far tested [4]. However, amyloid related imaging abnormities (ARIA), including edema (ARIA-E) and microhemorrhage (ARIA-H) are adverse effects associated with anti-amyloid immunotherapies and these risks are elevated in elderly people with cerebrovascular disease [5]. Thus, novel preventative and therapeutic approaches are urgently needed, particularly with respect to the vascular contributions to AD.

The importance of the cerebrovasculature in AD is underscored by the observation that up to 90% of AD autopsy cases have evidence of cerebral small vessel disease, including vascular microbleeds, vascular degeneration and deposition of Aβ in the vascular wall, a condition known as cerebral amyloid angiopathy (CAA) [6]. Sporadic CAA is also present in 10–40% of elderly brains [7]. Cerebrovascular pathologies precede other pathophysiological conditions and are believed to exacerbate or even initiate AD and neuroinflammation [8], perhaps by reflecting an age-related decline in the ability to clear Aβ from the brain by multiple pathways involving the cerebrovasculature [9]. Additionally, multiple cardiovascular risk factors such as hypertension, type II diabetes and dyslipidemia increase AD risk [10, 11] and a favourable cardiovascular health or management of cardiovascular risk factors, especially at midlife, may attenuate dementia risk decades later [12–14]. The clinical precedence of systemic factors that can affect AD pathogenesis raises the possibility that promoting vascular resilience may be an attractive preventative or therapeutic approach [15, 16].

High density lipoprotein (HDL) is a circulating lipid carrier that is best known for its pivotal role in reverse cholesterol transport, which is the process by which excess cholesterol is removed from cells and transported to the liver for excretion into bile [17]. HDL also has potent anti-thrombotic, anti-oxidant, anti-inflammatory and cytoprotective functions [18], all of which may affect AD. There is epidemiological evidence that AD risk can be attenuated by HDL levels [19]. Specifically, apolipoprotein (apo)A-I, the major HDL-associated protein, positively correlates with cognitive scores [20] and high serum HDL-cholesterol (> 55 mg/dl) in cognitively normal elderly individuals associates with reduced AD risk (hazard ratio (HR) 0.4) after adjusting for *APOE* genotype and vascular risk factors [21]. In mouse models of amyloidogenesis, deficiency of apoA-I, which leads to low HDL levels, is reported to selectively exacerbate CAA and cerebrovascular inflammation [22, 23], although other groups recently reported either no effect [24] or the opposite result [25]. Conversely, genetic apoA-I overexpression reduces CAA and neuroinflammation [26], and delivery of recombinant HDL or apoA-I Milano into the systemic circulation acutely decreases soluble brain Aβ levels and leads to long-lasting lowering of CAA and neuroinflammation, respectively [27, 28]. Importantly, although lipoprotein composition is generally similar between mice and humans [29], circulating lipids are mainly carried by HDL in rodents whereas they are mainly carried on low density lipoproteins (LDL) in humans, which may limit the translational relevance of some animal model studies [30].

We previously described a new human in vitro model of three-dimensional (3D) perfusable bioengineered vessels to study CAA and Aβ-associated vascular inflammation [31, 32]. In this study we use this vascular model to define several mechanisms by which HDL reduces Aβ accumulation in the engineered vascular wall and attenuates Aβ-induced vascular inflammation. First, the anti-CAA and anti-inflammatory functions of HDL are mediated by distinct mechanisms. HDL prevents Aβ vascular accumulation independently of its primary receptor, scavenger receptor (SR)-BI, in contrast to the previously described SR-BI-dependent mechanism by which HDL prevents Aβ-induced monocyte binding [32]. Second, delving deeper into HDL’s anti-CAA functions, we define four distinct pathways that HDL uses to attenuate Aβ accumulation, namely: i) altering Aβ binding to collagen-I, ii) forming a complex with Aβ that maintains its solubility, iii) diminishing collagen-I protein levels produced by smooth muscle cells (SMC), and iv) attenuating Aβ uptake into SMC that is associated with reduced low density lipoprotein receptor-related protein (LRP)1 levels. We also define a specific subfraction of HDL that may be particularly relevant to AD. Although only ~ 6% of circulating HDL lipoprotein particles contain apoE [33], it is this fraction of apoE-containing HDL (HDL-apoE) that appears to have the most potent effects on vascular amyloid deposition. ApoE is a major genetic risk factor for sporadic AD, where, in the brain, apoE is produced by astrocytes, microglia, pericytes and stressed neurons and contributes to AD pathogenesis by modulating multiple pathways, including but not limited to Aβ metabolism, tauopathy, and neuroinflammation [34]. Our observations provide new evidence that peripheral apoE, particularly the apoE found on a small fraction of circulating HDL lipoprotein particles, may play a greater role in AD pathogenesis than previously recognized.

## Methods

### Cells, lipoproteins and bioengineered tissues

Experiments with human cells were conducted under an approved clinical protocol (UBC Clinical Ethics Research Board H13–02719) after obtaining written informed consent. Human umbilical vein endothelial cells (EC) and human umbilical vein myofibroblasts (SMC) were isolated as described [31]. Briefly, EC were isolated using the collagenase (2 mg/ml, Collagenase A, Roche) instillation method and expanded in complete endothelial growth medium (EGM™-2) (LONZA Inc., supplemented with vascular endothelial growth factor (VEGF), human recombinant insulin-like growth factor-1 (hrIGF-1), human epidermal growth factor (hEGF), amphotericin-B, hydrocortisone, ascorbic acid, heparin, and 2% foetal bovine serum (FBS)) up to passage 8. SMCs were isolated by mincing the umbilical vein into small pieces (~ 2–3 mm) and expended in Advanced DMEM (Gibco) supplemented with 1% L-glutamine (Gibco), 0.05% Pen/Strep (Gibco) and 10% heat inactivated FBS (Gibco) up to passage 10. The RAW 264.7 murine macrophage line was purchased from ATCC and grown in DMEM (Gibco) containing 10% heat inactivated FBS, 1% L-glutamine and 0.05% Pen/Strep. Human primary astrocytes (Sciencell) were grown in complete astrocyte media containing 2% FBS (Sciencell) up to passage 6. Experiments with human blood were conducted under an approved clinical protocol (UBC Clinical Ethics Research Board H14–03357) and upon receipt of written informed consent. HDL (density 1.063–1.21 g/mL) and a mixture of plasma lipoprotein classes (very low density lipoprotein; vLDL, intermediate density lipoprotein; IDL and low density lipoprotein; LDL) (density < 1.063 g/mL) were isolated by KBr gradient ultra-centrifugation from blood of healthy volunteers and tested by SDS-PAGE as described [35] or purchased (Lee Biosource). Depletion or enrichment of apoE in HDL was performed as described [36]. Briefly, HDL was loaded on an apoE immunoaffinity column (Academy Biomedical) and incubated overnight at 4 °C before collecting the unbound fraction (apoE-depleted HDL). The column was then washed four times with PBS before eluting the bound fraction of HDL enriched in apoE (HDL-apoE) using 3 M NaSCN. Collected fractions were then extensively dialyzed against 400 mM NaCl and 1 mM EDTA.

HDL was fluorescently labeled using Alexa-647 (Invitrogen) or Atto-594 (Sigma Aldrich) following the manufacture’s guidelines and stored for a maximum of 1 month at 4 °C until use. Frozen brain tissues (cortex Brodmann area 9) from AD patients (Braak stage 4) or cognitively normal controls were provided by the Harvard Brain Tissue Resource Center under the approved UBC protocol (C04–0595) and maintained at − 80 °C until processing.

### Fabrication of engineered vessels

**Fig. 1:**
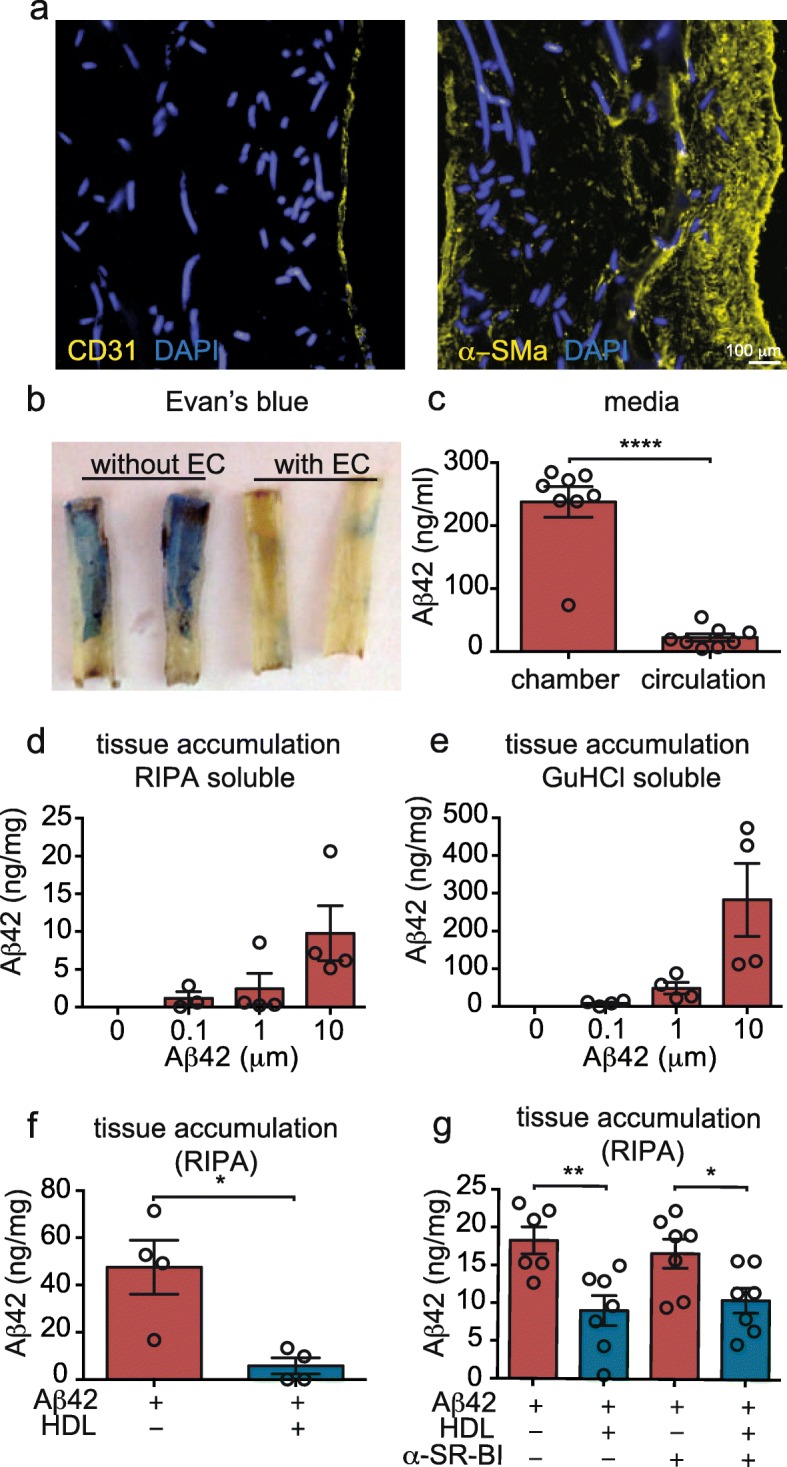
Human in vitro bioengineered vessels model CAA. **a** Immunofluorescent staining for CD31 confirms the formation of an endothelial monolayer surrounded by several layers of SMC stained for α-SM actin. **b** Evans blue staining confirms a tight endothelial barrier 10 days after endothelialisation. In (**a**) and (**b**), autofluorescence of the scaffold material is visible in the DAPI channel. **c** After injecting 1 μM of Aβ42 in the antelumen (tissue chamber), tissue chamber and circulation media were collected and Aβ42 was quantified by ELISA. **d-e** 24 h after injection of the indicated Aβ42 concentration into the tissue chamber, tissues were lysed in RIPA buffer before solubilizing the pellet in GuHCl followed by Aβ42 quantification using ELISA. **f** After injecting 1 μM of Aβ42 in the tissue chamber, 200 μg/mL of HDL was circulated through the lumen. After 24 h, Aβ42 accumulation was measured as above. **g** A blocking antibody against the HDL binding protein SR-BI was circulated in the presence of 200 μg/mL of HDL or BSA through the lumen after injecting Aβ42 into the tissue chamber. Aβ42 accumulation was measured as above. Points in graphed data represent individual bioengineered vessels, bars represent mean, error bars represent ±SEM and analysed by Student’ t-test or one way ANOVA *P < 0.05, **P < 0.01, ***P < 0.001 and ****P < 0.0001

Bioengineered vessels were fabricated using a dynamic, semi-pulsatile flow bioreactor system as described, illustrated in Sup. Fig. 1 [31, 32]. Briefly, tubular biodegradable scaffolds (length 1.5 cm and inner diameter 2 mm) were produced from non-woven polyglycolic acid (PGA, Biomedical Structure) meshes (thickness: 1 mm and density: 70 mg/cc) dip-coated with a 1.75% (w/w) polycaprolactone (PCL) (80 kDa, Sigma Aldrich)/tetrahydrofuran (THF, Sigma Aldrich) solution and externally coated with a 10% PCL/THF (w/w) solution. For bipartite vessels that resemble leptomeningeal arteries, ethanol-sterilized scaffolds were seeded with SMCs (2-3 × 10^6^ cells/cm^2)^ on the inner surface of the scaffold using fibrin gel (fibrinogen 10 mg clottable protein/ml PBS and thrombin 100–10 mU/ml PBS) as a cell carrier. After 3–4 days under static conditions, vascular tissues were exposed to dynamic flow. The flow of nutrient medium (AdvDMEM supplemented with 10% FBS, 1% L-glutamine, 0.05% Pen/Strep and 1.5 mM L-ascorbic acid (Sigma Aldrich)) was directed through the lumen of the vascular tissue to mimic blood flow for 7 days. HUVEC (1–1.5 × 10^6^ cells/cm^2^) were then seeded on the inner surface of the lumen and tissues were maintained first in static conditions for 5 days in EGM™-2 supplemented as above but with 10% FBS. After the static phase, vascular tissues were placed back in the bioreactor for 10 additional days. For tripartite tissues that resemble cortical penetrating arteries, astrocytes (1 × 10^6^ cells/cm^2^) were seeded on the antelumen of the vessel surface using fibrin as cell carrier 4–5 days after seeding EC and directly placed under flow condition for 10 days with complete astrocyte media in the tissue chamber and EGM™-2 supplemented as above with 10% FBS perfused through the lumen for 10 days. The vascular structure was evaluated by immunofluorescence and assessing the integrity of the endothelium using Evans blue as described [37] (Figs. 1a, b and 5a).

### Preparation of Aβ peptides

Recombinant Aβ42 (California Peptide, USA) or FITC-Aβ42 peptides (BACHEM, Switzerland) were dissolved in hexafluoroisopropanol (HFIP) to a concentration of 1 mM as described [32]. HFIP was removed by evaporation overnight and dried stocks were stored at − 20 °C. On the day of the assay, soluble monomers were prepared by reconstituting the peptide film in DMSO to 5 mM, which was diluted further to 100 μM in RPMI containing 1% Pen/Strep but without FBS. 100 μl of Aβ solution was injected into the tissue chamber containing 900 μl of complete astrocyte media (Sciencell) to the desired concentration using a syringe. For Aβ42 oligomers and fibrils, after reconstitution in RPMI, Aβ42 was incubated at either 4 °C (oligomers) or 37 °C (fibrils) for 48 h before injection into the tissue chamber.

### Aβ42 accumulation in bioengineered vessels

After 10 days under flow conditions after endothelization, the indicated concentration of monomers, oligomers or fibrils Aβ42 was injected into the tissue chamber (antelumen) in the absence or presence of 200 μg of protein/mL of HDL, LDL or BSA (vehicle control) that was injected into the bioreactor circulation loop (Sup. Fig. 1). Tissues were maintained under flow conditions during the entire experiment. At the indicated times, 5 mm tissue rings were collected, mechanically crushed and lysed in RIPA buffer (10 mM Tris pH 7.4, 150 mM NaCl, 1.0% NP-40, 1.0% sodium deoxycholate, 0.1% SDS and cOmplete protease inhibitor with EDTA (Roche)). After homogenization, samples were centrifuged for 15 min at 14000 g at 4 °C and the RIPA soluble fraction was transferred to a new tube. 5 mM of guanidine (GuHCl, Sigma Aldrich) was added to the tissue pellet and incubated at room temperature overnight under constant agitation before centrifugation at 14000 g at 4 °C for 15 min. RIPA soluble and GuHCl soluble fractions were stored at − 20 °C until quantification. RIPA (soluble) and GuHCl (insoluble) fractions were diluted 10 and 20 times respectively to quantify Aβ42 using a commercial ELISA kit (KHB3482, Life Tech) and normalized to total protein concentration measured by BCA assay (Fisher). Media collected in the tissue or circulating chambers 24 h after Aβ42 injection was diluted 100 times with complete EGM-2 and Aβ42 levels were quantified as above.

### Tissue inhibitor experiments

After ten days under flow conditions after endothelization, the indicated concentration of 1 μM Aβ42 was injected into the tissue chamber (antelumen) in the absence or presence of antibodies against SR-BI (NB400–113 Novus, 1:500, RRID:AB_1291690), or RAGE (MAB11451, R&D Systems, 1:50 RRID:AB_2224344), or recombinant receptor associated protein (RAP, Oxford biome 1 μM) that was injected into the bioreactor circulation loop. Tissues were then analyzed as described above for Aβ42 accumulation or monocyte binding.

### FITC-Aβ42 binding to collagen-I

Black 96-well plates were coated with a solution of rat-tail collagen-I (Fischer) or with 10% BSA in PBS at 37 °C. After 16 h, wells were washed three times with PBS before adding 100 μl of FITC-Aβ42 (1 μM) in RPMI media with 200 mg/ml of HDL or BSA and placed back in humidified incubator at 37 °C and 5% CO_2_ for 24 h. Wells were then washed three times with PBS before excitation at 490 nm and measuring emission intensity at 520 nm using an Infinite M2000 Pro plate reader (Tecan).

### Histology and immunofluorescence

Bioengineered vessels were fixed in 4% paraformaldehyde (PFA) for 30 min, cryopreserved in 20% sucrose in PBS for a minimum of 1 h, embedded in 5% bovine skin gelatin (Sigma Aldrich) and 20% sucrose in PBS and stored at − 80 °C until processed. Human cortical samples were stored at -80 °C until processed. On the processing day, samples were mounted in optimal cutting temperature (OCT) matrix before being sectioned on a cryotome (Leica). Briefly, bioengineered vessels and human cortical tissues were sectioned at 20 μm, with object and chamber temperatures of − 30 °C (engineered tissues) or − 20 °C (cortex), and sections were stored at − 80 °C until staining. For staining, sections were placed at room temperature for 10 min and rehydrated in PBS for 10 min. Human brain sections were then fixed for 15 min at room temperature followed by one 0.5 M tris-HCL (pH 8) and two PBS washes. SMC in 2D culture were grown on sterile microscope cover glasses (Paul Marlenfeld GmbH &Co) in 24 well plates or chamber slides (Fisher). After the indicated culture time, cells were washed three times with PBS, fixed with 4% PFA and washed once with 0.5 M Tris-HCL (pH 8) and twice with PBS.

Tissue sections and SMC cultured on coverslips were stained for endothelial markers CD31 (Biolegend, WM59, RRID: AB_31432) and vWF (Sigma Aldrich, AB_259543), muscular marker α-SM actin (Sigma Aldrich, 1A4, RRID: AB_476856), astrocyte marker GFAP (Abcam, GA5, RRID: AB_880203), amyloid marker Aβ 1–16 (Biolegend, 6E10, RRID_AB2533200), extracellular matrix markers collagen-I (Abcam, RRID: AB_731684), collagen-IV (EMD Millipore, RRID:AB_2276457), laminin (Abcam, RRID:AB_476856), biglycan (Abcam, RRID: AB_1523212), versican (Abcam, RRID: AB444865) and heparan sulfate proteoglycan (Abcam, A76L, RRID: AB_2295402), lysosome LAMP1 (Abcam, RRID: AB_775978) or Wheat Germ Agglutinin (WGA, ThermoFisher) as described [31]. Briefly, after rehydration in PBS for sections and fixation for SMC, samples were blocked at room temperature in PBS containing 5% donkey serum and 1% BSA (Sigma Aldrich). After 30 min samples were incubated overnight at 4 °C in a humidified chamber with specific antibodies against CD31 (1:50), von Willebrand factor (1:200), α-SM-actin (1:200), GFAP (1:200), 6E10 (1:100), collagen-I (1:200), collagen-IV (1:200), laminin (1:200), versican (1:200), biglycan (1:200), heparan sulphate proteoglycan (1:200) and LAMP1 (1:100). After three additional PBS washes, samples were incubated for 60 min at RT with donkey anti-rabbit or donkey anti-mouse Alexa-488 or Alex-594 secondary antibodies (Invitrogen, 1:600). After three additional PBS washes, sections were mounted in Prolong Gold antifade containing DAPI (ThermoFisher Scientific) and imaged using an Axioscan inverted microscope or Axioscan inverted confocal microscope (Zeiss).

### SDS-PAGE, native-PAGE and Immunoblotting

SMC or whole tissues were lysed in RIPA buffer (defined above) and centrifuged for 10 min at 12,000 g at 4 °C. Total protein concentrations were quantified using a BCA assay. Equal amounts of total protein (25 μg) were separated by either SDS-PAGE or Native PAGE, followed by electrophoretic transfer to polyvinylidene fluoride (PVDF) membranes (Millipore). Membranes were blocked for 1 h using 5% skim milk powder in PBS containing 0.5% Triton X (PBST). Collagen-I (Abcam, RRID: AB_731684, 1:1000), ApoE (Cell Signaling, D7I9N, RRID:AB_2798191, 1:1000), ApoA-I (Brookwood Biomedical, RRID: AB_2801381, 1:2000), ApoA-II (Abcam, RRID: AB_447950, 1:5000), Aβ 1–16 (Biolegend, 6E10, RRID_AB2533200, 1:1000), LRP1 (Abcam, EPR3724, RRID: AB_2234877, 1:20000), RAGE (Abcam, RRID: AB_2242462, 1:1000), GAPDH (EMD Millipore, RRID:AB_2107445, 1:1000) and Actin (Abcam, ACTN05 (C4), RRID: AB_303668, 1:1000) were immunodetected by incubating in primary antibody for 16 h in blocking buffer at 4 °C. Membranes were washed extensively with PBST and incubated with anti-mouse, anti-goat or anti-rabbit (1:1000–10,000, Jackson ImmunoResearch) secondary antibody in blocking buffer. After 1 h, membranes were washed extensively with PBST, developed using enhanced chemiluminescence (ECL, Amersham) and imaged using ChemiDoc MP imager (Biorad). Band densitometry was quantified using image J and normalized to loading control, actin or GAPDH.

### Flow cytometry

SMC and EC were seeded in 6-well plates at a concentration of 300,000 and 500,000 cells per well, respectively. After two days in culture, cells were incubated with 0.1 μM of FITC-Aβ42 (Bachem) in the absence or presence of 200 μg/mL of HDL or BSA in respective growth media. After 3 or 24 h, cells were washed twice with PBS, detached using 0.25% trypsin for 5 min at 37 °C before adding RPMI to collect the cells. Floating cells were pelleted by centrifugation (300 g) for 5 min, washed four times with FACS buffer (PBS containing 2% FBS, 1 mM EDTA and 0.1% sodium azide), and suspended in a final volume of 200 μL of FACS buffer and counted immediately using a BD™ LSRII flow cytometer. Data were analysed using FlowJo software (RRID:SCR_008520).

### Gel-filtration chromatography

A Superdex 200-increase 10/300 column was equilibrated in filtered and degassed phenol red-free DMEM and 0.02% sodium azide overnight prior to loading of protein samples. HFIP treated Aβ42 was reconstituted in 100% DMSO prior to dilution in DMEM to a concentration of 100 μM or mixing with 1 mg/mL HDL or BSA. The sample was then centrifuged at 20,000 x g for 10 min before 100 μL of supernatant was injected onto the column and run at a flow rate of 0.4 mL/min. Following sample injection, 0.5 mL fractions were collected across the entire column volume of each run. Collected fractions were stored at − 20 °C prior to being processed by dot blot. Protein standards were run under the same conditions as above, and included alcohol dehydrogenase (150 kDa), BSA (66 kDa), carbonic anhydrase (29 kDa), and cytochrome c (12.4 kDa), all purchased from Sigma Aldrich. Gel-filtration fractions, collected as column eluents, were loaded onto nitrocellulose membranes through a round slot-format 96-well microfiltration apparatus (Biorad), then immunodetected and developed following the above immunoblotting protocol. Densitometry was performed with Image Lab (Biorad, RRID:SCR_001935).

### Cholesterol efflux

RAW 264.7 cells were seeded at 150,000 cells/well in 24-well plates and cultured for 24 h before labeling with 1 μCi/mL of ^3^H-Cholesterol (PerkinElmer Life Sciences) in DMEM containing 10% heat inactivated FBS, 1% Pen/Strep and 1% L-glutamine. After 24 h, labeled cells were washed and equilibrated in serum-free media for at least 60 min. Serum-free media was then added to the cells in the absence (NA, no acceptor) or presence of 50 μg/mL of HDL or 10 μg/mL of exogenous human serum delipidated apoA-I (a gift from CSL Behring, Switzerland) that was previously incubated for 24 h with 1 μM Aβ42 or DMSO at 4 °C. After 4 h at 37 °C, culture media was collected and cells were lysed with 0.1 M NaOH with 0.2% SDS, followed by incubation at room temperature for a minimum of 1 h. β-radiation in media and cell lysate samples were quantified by scintillation counting (PerkinElmer). The percentage cholesterol efflux was calculated as the total counts per minute (CPM) in the media divided by the sum of the CPM in the media plus in the cell lysate [38].

### Thioflavin-T quantification

Cell-free Thioflavin-T fibrillization assays were performed as described [32]. Briefly, 10 μM monomeric Aβ42 were incubated in 20 mM of Thioflavin-T in 150 mM NaCl and 5 μM of HEPES at pH 7.4, with and without 1 mg/mL of HDL, LDL or BSA, at 37 °C with 20 s of orbital shaking (3 mm amplitude) every 5 min in a black 96-well plate. Formation of fibrillar β-amyloid beta sheets over time was monitored by excitation at 440 nm and measuring emission intensity at 490 nm every 5 min up to 12 h in total using an Infinite M2000 Pro plate reader. Fibrillization curve were plotted using GraphPad Prism-5 software (RRID:SCR_002798), V50, final fluorescence and lag phase were measured as described [39].

### Statistics

Comparisons between groups were performed using Student T-test when comparing two groups or one-way ANOVA with Dunnett or Bonferroni post hoc tests when comparing multiple groups. Data were obtained from at least 3 independently generated bioengineered vessels or experiments and graphically represented as mean ± standard error of the mean (SEM). *P*-values of < 0.05 were considered statistically significant. All statistical analyses were performed using GraphPad Prism-5 software (RRID:SCR_002798) or SPSS (RRID:SCR_002865).

## Results

### Circulating HDL reduces vascular Aβ42 accumulation independently of SR-BI in a human in vitro vascular model of CAA

CAA is the accumulation of Aβ within leptomeningeal and cortical cerebral vessel walls. Although CAA is highly associated with the Flemish, Iowa and Dutch genetic mutations in amyloid precursor protein, sporadic CAA is present in 10–40% of non-cognitively declined elderly brains and in 80% of AD brains [7, 40]. To reproduce CAA in vitro, leptomeningeal-like vessels were fabricated by sequentially seeding primary human SMC and endothelial cells (EC) into a tubular scaffold. After 4 weeks under flow culture, immunohistochemical staining confirmed multiple layers of α-smooth muscle actin (α-SMA) positive cells on the inner side of the scaffold and a monolayer of CD31 positive EC lining the vascular lumen (Fig. 1a). Integrity of the endothelial barrier was demonstrated by injecting Evans blue into the circulation loop of the bioreactor and observing exclusion from engineered tissues after endothelization (Fig. 1b). CAA is modeled by injecting monomeric Aβ into the tissue chamber (antelumen; “brain side”) of the bioreactor at the indicated final concentrations to mimic Aβ produced by neurons that would be located around vessels (Sup. Fig. 1). Although Aβ40 is the predominant Aβ species in CAA, in this study we focused on Aβ42 because it is considered indispensable to initiate CAA lesions [41] and because we previously reported that HDL has a stronger protective effect against Aβ42 vascular deposition compared to Aβ40 [31]. Upon injection of Aβ42 in the antelumen chamber, we confirmed endothelial barrier integrity by measuring Aβ concentration by ELISA in both the antelumen chamber and in the circulating media 24 h after injection, and observed that Aβ42 levels in the antelumen chamber remained 11-fold more concentrated than in the circulation media (Fig. 1c). We also prepared RIPA and GuHCl fractions from tissues harvested 24 h after Aβ42 injection and, using ELISA, observed dose-dependent tissue accumulation of soluble and aggregated Aβ42, respectively (Fig. 1d-e).

Multiple epidemiological studies associate high levels of plasma HDL at midlife with reduced AD risk, and several laboratories, including ours, demonstrated that HDL reduces CAA and vascular inflammation using in vitro and AD rodent models [15, 27, 28, 31, 32]. However, the mechanisms by which HDL attenuates Aβ-induced inflammation and reduces CAA remain unknown. As lipoprotein metabolism differs substantially between mice and humans [30], we used our engineered human CAA model to provide mechanistic insights as to how human HDL affects CAA in human bioengineered vessels. We first used Aβ42 ELISA assays to confirm that circulating HDL (200 μg/mL) in the lumen reduced Aβ42-vascular accumulation by 8-fold in bioengineered vessels (Fig. 1f). Because SR-BI is the principal binding partner of HDL on EC [42] and we previously showed that blocking SR-BI annihilates HDL ability to reduce Aβ–induced monocyte binding to EC [32], we tested whether blocking SR-BI using a specific antibody eliminated HDL’s beneficial effects on Aβ accumulation in the vascular wall. However, blocking SR-BI did not alter HDL’s ability to reduce Aβ42 accumulation by HDL, showing that HDL’s anti-CAA activity is independent of SR-BI (Fig. 1g). Thus, the anti-CAA and anti-inflammatory effects of HDL against Aβ bifurcate along two major pathways; a SR-BI-independent pathway for CAA and a SR-BI-dependent pathway for Aβ-mediated endothelial inflammation.

### Reducing vascular Aβ42 accumulation is specific to HDL

**Fig. 2:**
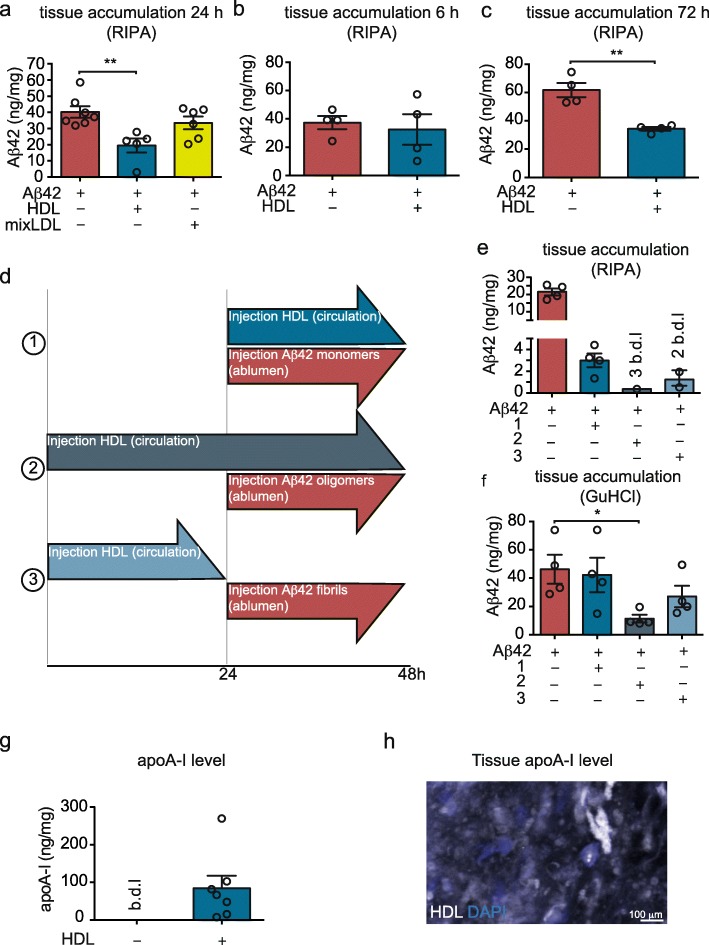
HDL, but not LDL, reduces Aβ42 accumulation in the bioengineered vessels by penetrating in the vascular wall. **a** Aβ42 monomers (1 μM) were injected into the tissue chamber concomitantly with circulating 200 μg/mL (total protein) of HDL or mixLDL (LDL, VLDL and ILDL) through the lumen. After 24 h, tissues were lysed in RIPA and Aβ42 was measured using ELISA. After injection of Aβ42 as above, HDL (200 μg/mL) was circulated through the lumen and Aβ42 accumulation was measured after 6 h (**b**) or 72 h (**c**). **d** Schematic representation of the experimental design for panels (**e**) and (**f**). Bioengineered vessels were treated with Aβ42 with or without HDL (1) or pre-treated with 200 μg/mL HDL for 24 h before injecting Aβ42 without (2) or with (3) washing. 24 h after Aβ42 injection, Aβ accumulation was measured in RIPA (**e**) or GuHCl (**f**) fractions. **g** HDL (200 μg/mL) was circulated through lumen of bioengineered vessels for 24 h before measuring apoA-I in vascular tissues using ELISA. **h** Alexa 633-labeled HDL (depicted in white) was circulated through the circulation of engineered vessel before, washing, fixing and imaging using confocal microscopy, representative image of three bioengineered vessels. Points in graphed data represent individual bioengineered vessels, bars represent mean, error bars represent ±SEM and analysed by Student’s t-test or one way ANOVA *P < 0.05, **P < 0.01, ***P < 0.001 and ****P < 0.0001

Previous studies have reported that HDL and associated apolipoproteins can alter Aβ aggregation in vitro [32, 43, 44], and here we show that a preparation consisting of low density lipoprotein (LDL), intermediate density lipoprotein (IDL) and very low density lipoprotein (VLDL) (density < 1.063 g/mL, mixLDL) also delays Aβ42 fibrillization using a cell-free Thioflavin-T fibrillization assay (Sup. Fig. 2a-d). We therefore tested if all of these plasma lipoproteins can reduce Aβ42 accumulation in bioengineered vessels through which 200 μg/mL (total protein) of either HDL (density: 1.063–1.21 g/mL) or the mixLDL fraction, isolated from the same healthy normolipidemic individuals, was circulated. As HDL reduced Aβ42 accumulation by 2-fold whereas mixLDL had no significant effect on tissue Aβ42 levels measured 24 h after injection, only HDL can protect from vascular Aβ accumulation (Fig. 2a). We confirmed that HDL did not reduce RIPA-soluble Aβ42 by shifting aggregates to the GluHCl-soluble fraction (Sup. Fig. 3a). HDL appears to act only on Aβ monomers, as it failed to reduce Aβ42 vascular accumulation after injecting either preformed oligomers or fibrils (Sup. Fig. 3b-c), and does not reduce Aβ42 accumulation when Aβ42 is injected prior to HDL treatment (Sup. Fig. 3d). As HDL had no effect on Aβ42 vascular accumulation when tested 6 h after Aβ42 injection (Fig. 2b) but maintains the significant 2-fold reduction of Aβ42 accumulation up to 72 h (Fig. 2c), we hypothesised that HDL requires time, possibly to prime the vascular wall, to reduce Aβ accumulation.

To test this hypothesis, we compared the effect of HDL circulated prior to or concomitantly with injection of Aβ42 (Fig. 2d), and observed a stronger reduction in the RIPA-soluble Aβ42 fraction when HDL was circulated 24 h prior to injecting Aβ42, with three out of four tissues below the detection limit of the ELISA, compared to HDL/Aβ42 co-treatment (Fig. 2e). Interestingly, we observed that pre-treatment with HDL also reduced Aβ42 accumulation in the GuHCl-soluble fraction by 4-fold compared to vehicle control, respectively (Fig. 2f). We then tested whether washing out HDL after 24 h of circulation affected vascular Aβ42 accumulation. Interestingly, Aβ42 accumulation in the RIPA fraction was reduced to a similar extent as when HDL was maintained in the circulation, with two out of four samples below the detection limit of the ELISA (Fig. 2e). Aβ42 accumulation in the GuHCl-soluble fraction tended to be reduced by 1.7-fold compared to vehicle control after HDL wash-out (Fig. 2f). Together, these results suggest that HDL primes the vasculature to resist Aβ42 accumulation, potentially by some component(s) of HDL penetrating the vascular wall.

### HDL enters the vasculature and reduces Aβ42 uptake by vascular SMC

The presence of HDL-associated apolipoproteins in the wall of human cerebral arteries with and without CAA has been demonstrated using both proteomic [45] and immunohistochemistry methods, and show that apoA-I, apoJ and apoE co-localize with CAA in human cerebral arteries [46]. Although apoE and apoJ are both expressed in the brain and found in the peripheral circulation, apoA-I is not expressed within the brain as it is secreted exclusively from hepatocytes and enterocytes [47]. As expected, bioengineered vessels have no detectable apoA-I when HDL is absent, and have an average of 84 ng of apoA-I per mg of total tissue protein after circulating HDL for 24 h (Fig. 2g), demonstrating that at least the apoA-I moiety on HDL particles can enter the engineered vascular wall. Further, fluorescently labeled HDL (fluorescent dye bound to protein moiety) accumulates in bioengineered vessels (Fig. 2h).

**Fig. 3:**
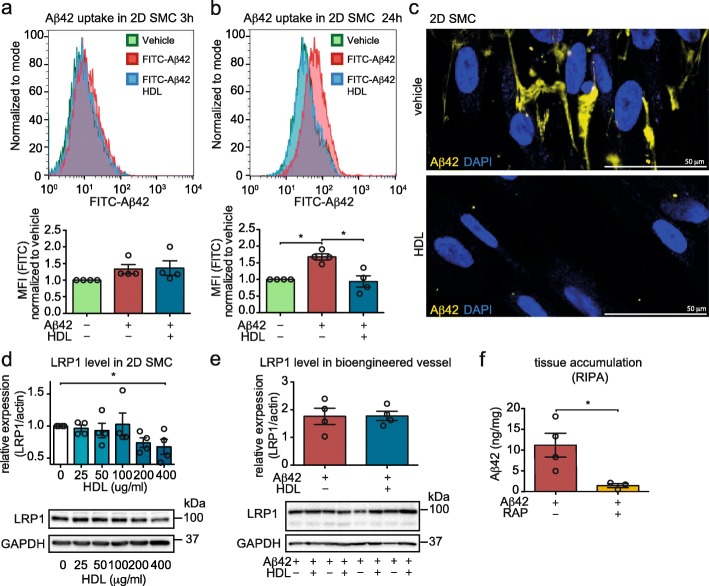
HDL reduces Aβ42 uptake and LRP1 levels in SMC and blocking LRP1 with RAP reduces Aβ42 accumulation in bioengineered vessels. SMC were grown as 2D monolayers for 2 d before treating with 1 μM FITC-Aβ42 without or with 200 μg/mL of HDL for 3 h (**a**) or 24 h (**b**) before dissociating the cells and counting using flow cytometry. **c** SMC were grown in 2D culture for 3 d before treating with FITC-Aβ42 (5 μM, yellow) with 200 μg/mL of HDL or BSA for 24 h and imaged using microscopy. **d** SMC were grown for 3 days in 2D culture were treated with the indicated HDL concentration. LRP1 protein levels were measured by Western blot and normalized to GAPDH. **e** Aβ42 monomers (1 μM) were injected into the tissue chamber concomitantly with circulating 200 μg/mL of HDL or BSA through the lumen. After 24 h, tissues were lysed in RIPA. LRP1 protein levels were measured in bioengineered vessels using Western blot and normalized to GAPDH. **f** Bioengineered vessels were treated with 1 μM Aβ42 with or without recombinant RAP in the circulation media to block LRP1. After 24 h, tissues were lysed in RIPA and Aβ42 accumulation was measured by ELISA. Points in graphed data represent individual bioengineered vessels, bars represent mean, error bars represent ±SEM and analysed by Student’s t-test or one way ANOVA *P < 0.05

Our previous studies demonstrated that HDL reduces Aβ42 uptake into EC [32]. Here, we used flow cytometry experiments to show that HDL also blocks Aβ42 uptake into SMC, but with a kinetic difference compared to EC uptake. Specifically, SMC cultured in regular 2D tissue culture plates take up FITC-Aβ42 after 24 h but not after 3 h, indicating a slower uptake of Aβ42 into SMC compared to EC (Fig. 3a-b and Sup. Fig. 4a-b). Furthermore, after 24 h, the median fluorescent signal was increased only by 1.7-fold in SMC but was elevated by 9.2-fold in EC suggesting less overall uptake of Aβ42 into SMC compared to EC (Fig. 3a-b and Sup. Fig. 4a-b). After 24 h in the presence of HDL, FITC-Aβ42 uptake remains at background levels, demonstrating that HDL blocks Aβ42 uptake in SMC (Fig. 3a-b). These results were further confirmed by microscopy (Fig. 3c). We also showed that the reduced fluorescent intensity of FITC-Aβ42 after HDL treatment was not due to increased co-localization with the lysosomal marker LAMP1, which would suggest enhanced degradation after HDL treatment (Sup. Fig. 4c).

We next assessed the role of the low density lipoprotein related protein (LRP)1, the major Aβ binding partner in SMC [48], by measuring LRP1 protein levels after HDL treatment. Although HDL reduced LRP1 levels after 24 h in SMC in regular tissue culture plates (Fig. 3d), it did not affect LRP1 levels in bioengineered vessels at the 24 h time point (Fig. 3e), However circulating recombinant receptor associated protein (RAP) through the lumen of bioengineered vessels to block LRP1 led to a significant 8-fold reduction in Aβ42 accumulation, similar to the effects observed with HDL (Fig. 3f). Taken together these results suggest that blocking Aβ uptake into SMC via LRP1 reduces vascular Aβ42 accumulation in bioengineered vessels.

Several experiments were performed to determine if HDL affects “blood-to-brain” transport of Aβ. Entry of Aβ from the periphery into the brain is mediated by the receptor for advanced glycation endproducts (RAGE) [49] and has been reported to cause both parenchymal and CAA pathologies [50, 51]. Although we observed that RAGE levels in engineered tissues were increased 2-fold upon HDL treatment, approaching significance (Sup. Fig. 5a), Aβ accumulation is reduced in the presence of HDL. We also tested whether HDL blocks accumulation of circulating Aβ42 by directly comparing Aβ42 accumulation in bioengineered vessels when Aβ42 was injected in the antelumen chamber or into the circulation loop at a final concentration of 1 μM. As expected, after 24 h, Aβ42 levels were higher in the chamber into which it was injected. Specifically, injection of Aβ42 into the tissue chamber resulted in 5-fold higher Aβ42 levels in the tissue chamber media compared to the circulating media (Sup. Fig. 5b), whereas injection into the circulation loop resulted in 5.5-fold higher Aβ levels in the circulating media compared to the tissue chamber media (Sup. Fig. 5c), demonstrating that Aβ42 does not diffuse across the bioengineered vessels. After 24 h, we observed that vascular accumulation of Aβ42 was 3-fold higher when Aβ42 was injected into the antelumen chamber compared to the circulation loop, and that HDL only reduced Aβ42 vascular tissue accumulation when Aβ42 was injected in the antelumen chamber (Sup. Fig. 5d). We further investigated if blocking peripheral Aβ42 uptake using a RAGE antibody altered Aβ42 accumulation and found no effect (Sup. Fig. 5e).

### Aβ co-localizes with collagen-I in human post-mortem CAA and HDL reduces collagen-I expression in monolayer SMC cultures

**Fig. 4:**
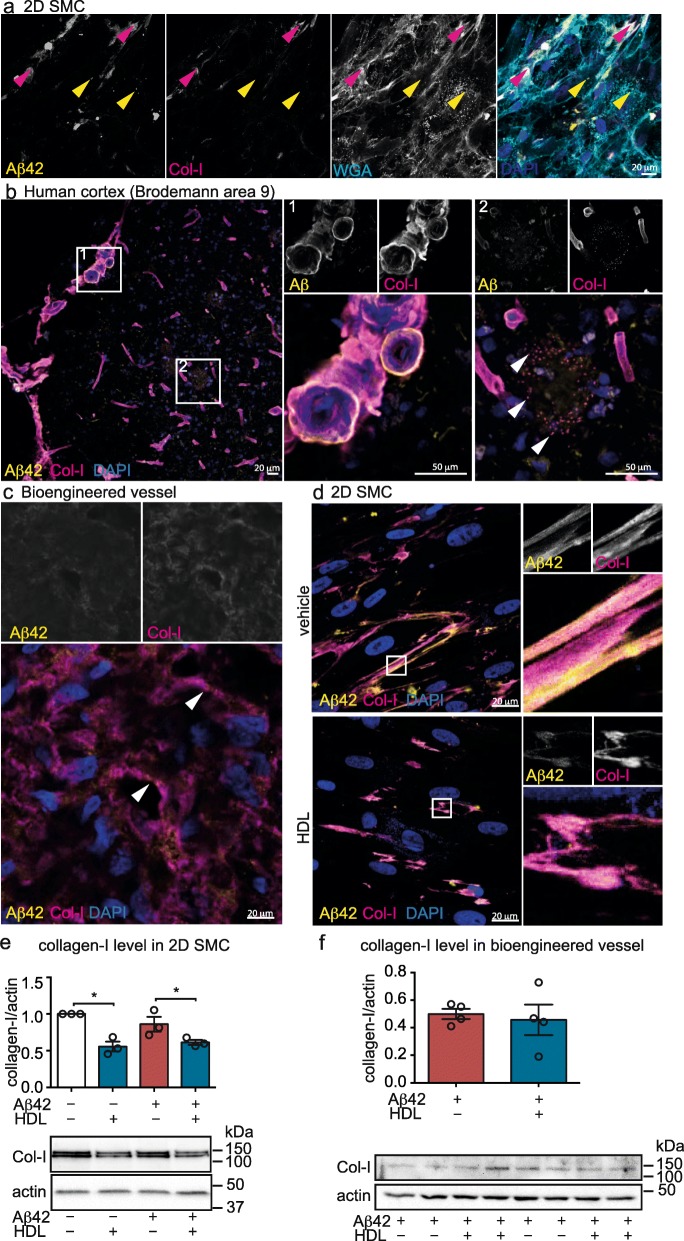
Aβ42 binds to collagen-I in the vascular wall of post-mortem human brain and in bioengineered vessels and HDL reduces collagen-I level in SMC. **a** SMC were grown for 1 d in chamber slides before treating with 0.5 μM FITC-Aβ42 (yellow) and 200 μg/ml HDL. After 24 h, cells were fixed and cell membranes were stained using wheat germ agglutinin (WGA, magenta) and an antibody against collagen-I (cyan) was used to stain the ECM. Yellow arrows show intracellular Aβ42 and magenta arrows show collagen-I/Aβ42 co-localization. **b** Human cortex (Brodmann area 9) from four donors was stained for Aβ using 6E10 (yellow) and collagen-I (magenta) and expanded views of vascular (1) and cortical (2) Aβ fibrillar plaques are presented on the right. **c** Bioengineered vessels treated with Aβ42 monomers for 24 h were stained for collagen-I (yellow) and Aβ (6E10, magenta). White arrows show co-localization of collagen-I and Aβ42. **d** SMC were grown on 2D coverslips for 24 h before incubating with 0.5 μM FITC-Aβ42 (yellow) and 200 μg/mL HDL. SMC were then fixed and stained for collagen-I (magenta). Details of collagen-I fibres and Aβ42 co-localization are presented on the right. **e** SMC were grown for 24 h in 2D culture before treating with 1 μM Aβ42 with or without 200 μg/mL HDL. After 24 h, cells were lysed, collagen-I expression was measured by Western blot and normalized to actin levels. **f** Bioengineered vessels were treated with 1 μM Aβ42 and 200 μg/mL HDL as above. After 24 h, bioengineered vessels were lysed in RIPA and collagen-I levels were measured by Western blot and normalized to actin levels. All images are representative of at least 3 individual experiments or 4 donors and were imaged using confocal microscopy. Points in graphed data represent individual bioengineered vessels, bars represent mean, error bars represent ±SEM and analysed by Student’s t-test *P < 0.05

As treating 2D cultures of SMC with Aβ42 leads to a staining pattern that does not resemble the vesicular like-shape of typical intracellular staining (Fig. 3c), we next investigated whether Aβ42 is taken up by SMC or whether it remains bound to the cell membrane or extracellular matrix (ECM) produced by SMC. Fluorescent analysis using fluorochrome-coupled wheat germ agglutinin (WGA) to delineate the cell membrane and anti-collagen-I to stain the ECM showed that the Aβ42 signal was both extracellular (magenta arrows) and intracellular (yellow arrows) (Fig. 4a). We further used immunofluorescence staining to determine whether Aβ co-localizes with vascular ECM proteins in post mortem human brain (cortex Brodmann area 9). We found that Aβ co-localized with collagen-I in the vascular wall (Fig. 4b1) but not in parenchymal plaques (Fig. 4b2) and observed punctuate staining in the periphery of the plaques (arrows) in half of the patient brains tested. Vascular Aβ also partially co-localized with collagen-IV but not with laminin, which was located closer to the lumen of the vessels (Sup. Fig. 6a-b). Finally, we tested whether Aβ co-localizes with proteoglycans such as biglycan (Sup. Fig. 6c), heparan sulphate proteoglycan 2 (HSP2)/perlecan (Sup. Fig. 6d) and versican (Sup. Fig. 6e) and found that only vascular Aβ co-localized with these proteoglycans. We then investigated Aβ42 co-localization with the ECM in engineered vessels and found that Aβ42 co-localized with collagen-I (white arrows) (Fig. 4c).

Because a previous study suggested that HDL reduces collagen-I levels in mouse aortic SMC [52], we evaluated FITC-Aβ42 and collagen-I levels in sub-confluent SMC cultured in regular 2D plates in the absence or presence of HDL and observed lower signals for both FITC-Aβ42 and collagen-I staining 24 h after HDL treatment (Fig. 4d). We confirmed these results by Western blotting to show that HDL reduced collagen-I protein levels in SMC by 2-fold compared to vehicle control, whereas Aβ42 did not alter collagen-I levels (Fig. 4e). Interestingly, collagen-I levels were not altered in bioengineered vessels after 24 h HDL treatment (Fig. 4f), suggesting that a reduction in collagen-I levels is unlikely to explain the mechanism of action of HDL on accumulation of Aβ42 in bioengineered vessels at acute time points.

### HDL reduces Aβ42 binding to collagen-I by forming a complex

**Fig. 5:**
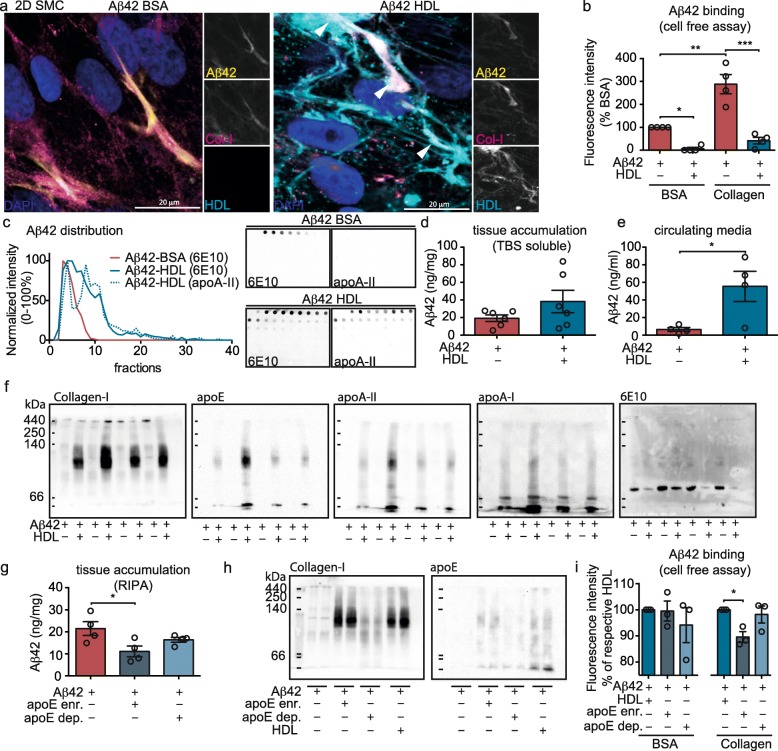
HDL binds to both collagen-I and to Aβ42 to form a complex and increases Aβ transport through the bioengineered vessel wall primarily by HDL-apoE particles. **a** SMC were grown for 3 d on 2D coverslips before incubating with 0.5 μM FITC-Aβ42 (depicted as yellow) and 200 μg/ml Alexa-633 labeled HDL (depicted as cyan). After 24 h, cells were fixed and stained for collagen-I (magenta) before imaging by confocal microscopy. White arrows show co-localization of Aβ42, HDL and collagen-I. **b** Black 96 well-plates were coated either with 50 μg/mL rat-tail collagen-I or 10% BSA as protein control for 24 h before incubating with a solution of 1 μM of FITC-Aβ42 with or without 200 μg/mL of HDL. After 24 h and extensive PBS washes, fluorescence representing bound Aβ42 was measured at 520 nm, excitation 490 nm. **c** 1 μM Aβ42 were incubated either with 200 μg/mL of HDL or BSA for 24 h at 37 °C before gel-filtration chromatography separation. Fractions were dot blotted and immunodetected for Aβ (6E10) or HDL (apoA-II). The intensity of each fraction on the dot blot was quantified, normalized between 0 and 100% and graphed on the left panel. The right panel shows a representative dot blot used for quantification. **d** 1 μM Aβ42 was injected in the tissue chamber of bioengineered vessels and 200 μg/mL of HDL was circulated through the lumen. After 24 h, tissues were homogenized in TBS to collect soluble Aβ, which was quantified in the tissue (**c**) or circulating media (**d**) by ELISA. **f** 1 μM Aβ42 was injected in the tissue chamber of bioengineered vessels and 200 μg/mL of HDL was circulated through the lumen. After 24 h vessels were lysed in RIPA and protein distribution was analysed using non-denaturing native blot probed for collagen-I, apoE, apoA-II, apoA-I and Aβ (6E10). **g** HDL was enriched for or depleted of apoE using an apoE immunoaffinity column. Bioengineered vessels were then treated with either fraction (200 μg/mL) in the presence of 1 μM Aβ42 as above before measuring Aβ deposition in the RIPA-soluble fraction. **h** RIPA lysates from engineered vessels were then analysed using non-denaturing native blot probed for collagen-I and apoE. **i** Black 96-well plates were coated either with 50 μg/mL rat-tail collagen-I or 10% BSA for 24 h before incubating with a solution of 1 μM of FITC-Aβ42 with or without 200 μg/ml of total HDL, apoE-depleted HDL or apoE-enriched HDL. After 24 h and extensive PBS washes, fluorescence was measured at 520 nm, excitation 490 nm. FPLC data and Western blots are representative of at least 3 individual experiments. Points in graphed data represent individual bioengineered vessels, bars represent mean, error bars represent ±SEM and analysed by Student’s t-test or one way ANOVA *P < 0.05, **P < 0.01, ***P < 0.001 and ****P < 0.0001

As we previously reported that the staining pattern of HDL in the vascular wall is similar to the ECM pattern [37], we tested whether HDL might compete with and prevent Aβ42 binding to collagen-I fibres. First, we cultured SMC for 3 days in 2D chamber slides to allow time for ECM deposition. SMC were then incubated with FITC-Aβ42 with or without fluorochrome-labeled HDL before immunostaining for collagen-I. We confirmed that FITC-Aβ42 bound to collagen-I and demonstrated that HDL partially co-localized with collagen-I (arrows) (Fig. 5a). We next used a cell-free assay where 96-well plates were coated with either rat-tail collagen-I or BSA as a control and incubated with FITC-Aβ42 with or without HDL for 24 h. We showed that Aβ42 binding to collagen-I was 3-fold higher than the BSA control and that HDL reduced Aβ42 binding to both BSA and collagen-I by 11.8- and 7-fold, respectively (Fig. 5b). These data suggest that HDL may help maintain Aβ42 in solution not only by competing for binding to collagen-I but also by forming a complex with Aβ. We confirmed the formation of an Aβ42/HDL complex using gel-filtration chromatography and fraction analysis in a dot blot immunoassay. After incubating Aβ42 with either HDL or BSA as a protein control, mixtures were separated by size exclusion and fractions were dotted onto nitrocellulose and probed for Aβ and apoA-II, a representative HDL protein. The UV spectrum analysis did not reveal major differences between HDL with or without Aβ42 (Sup. Fig. 7a-c). Conversely, densitometry analysis of the dot blots demonstrated that the Aβ42 elution distribution range was increased after incubation with HDL and that Aβ42 co-distributed with HDL fractions (Fig. 5c). Importantly, untreated HDL fractions were negative for Aβ42 (Sup. Fig. 7a). We further confirmed the formation of a complex by measuring a subtle but significant decrease in cholesterol efflux from RAW264.7 macrophages to HDL + Aβ42 compared to HDL alone (Sup. Fig. 7d). Incubating lipid-free apoA-I with Aβ42 also reduced cholesterol efflux activity compared to apoA-I alone, suggesting that the Aβ42/HDL complex might be preferentially associated with the protein moiety of HDL (Sup. Fig. 7e). Aβ42 alone did not alter basal cholesterol efflux activity (Sup. Fig. 7f).

We then hypothesised that if HDL acts to maintain Aβ42 solubility in the vascular wall, we should observe an increase in Aβ42 levels in vascular extracellular fluid (TBS soluble fraction) and enhanced Aβ42 recovery in the circulating media after HDL treatment due to more efficient transport through the vascular wall. Although not significant, the concentration of Aβ42 in the TBS-soluble fraction was increased by 2-fold 24 h after HDL treatment, while Aβ42 levels in circulating media were significantly increased by 8.8-fold after HDL treatment (Fig. 5d-e). These observations strongly suggest that HDL reduces Aβ accumulation by increasing “brain-to-blood” transport of soluble Aβ42 through the vascular wall to the circulation.

### ApoE-containing HDL particles reduce vascular Aβ42 accumulation more effectively than apoE-depleted HDL

As HDL does not affect total collagen-I levels in engineered vessels after 24 h yet HDL binds to collagen-I, we next used Native-PAGE and immunoblotting to determine if HDL affects collagen-I migration. After circulating HDL through the vessel for 24 h, collagen-I migrated as species of ~ 100 kDa, which is smaller than the typical triple helix structure of collagen-I that is too big to enter the acrylamide stacking gel (~ 520 kDa) [53]. Interestingly, these smaller collagen-I species co-migrated through Native-PAGE gels with apoE-HDL and apoA-II-HDL particles, whereas apoA-I-HDL particles have a smaller size (Fig. 5f). We then hypothesised that the apolipoprotein composition of HDL may affect Aβ42 vascular accumulation. We prepared apoE-depleted or apoE-enriched HDL using an apoE immunoaffinity column. When normalized to apoA-I levels, apoE-depleted HDL had 4-fold lower and apoE-enriched HDL had 30-fold higher apoE levels than total plasma HDL (not shown). After circulating 200 μg/mL (total protein) of these HDL fractions (corresponding to equivalent apoA-I concentrations) through the lumen of bioengineered vessels we observed that apoE-depleted HDL reduced Aβ42 accumulation only by 1.3-fold, whereas apoE-enriched HDL caused a significant 2-fold reduction (Fig. 5g). Further, only apoE-enriched HDL affected the migration of collagen-I through Native-PAGE gels (Fig. 5h). Finally, we used the cell-free collagen-binding assay to show that apoE-enriched HDL reduced Aβ42 binding to collagen more effectively than apoE-depleted HDL (Fig. 5i).

### Bioengineered tripartite vessels with astrocytes exhibit lower Aβ42 accumulation, native apoE in the tissue chamber media, and reduced Aβ42 binding to collagen-I

**Fig. 6:**
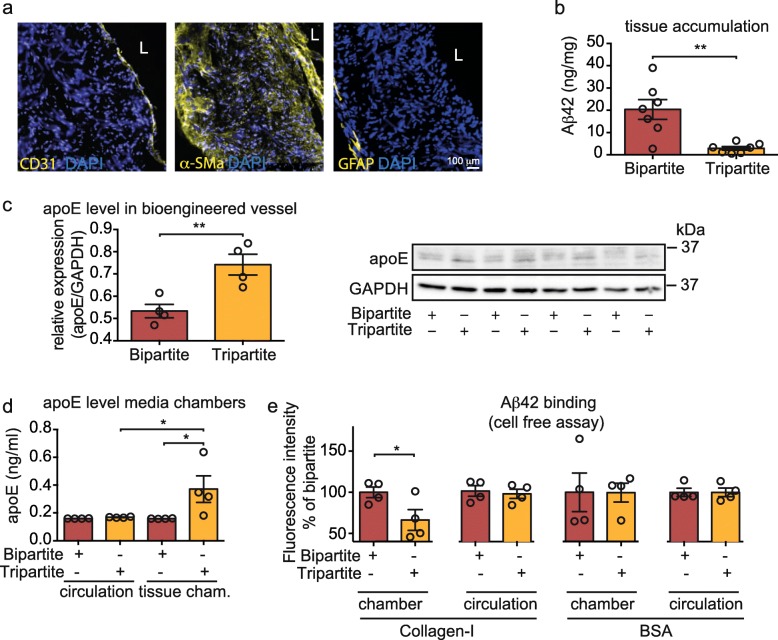
Bioengineered vessels with astrocytes have reduced Aβ42 accumulation compared to bioengineered vessel lacking astrocytes and astrocyte-derived apoE in tissue chamber media reduces Aβ42 binding to collagen-I. **a** Immunofluorescent staining confirms the formation of an endothelial monolayer (CD31) surrounded by several layers of SMC (α-SM actin) with astrocytes genotyped as *APOEε3/3* on the antelumen (GFAP). **b** 1 μM Aβ42 was injected into the tissue chamber of bioengineered vessels without (bipartite) or with astrocytes (tripartite). After 24 h, tissues were lysed in RIPA and Aβ42 accumulation was quantified using ELISA. **c** apoE levels in bipartite or tripartite bioengineered vessels were measured in RIPA lysate by immunoblot and normalized to GAPDH. **d** Astrocyte-secreted apoE levels in the circulation and chamber media were measured by ELISA. **e** Black 96-well plates were coated either with 50 μg/mL rat-tail collagen-I or 10% BSA for 24 h before incubating with a solution of 1 μM of FITC-Aβ42 in conditioned media from the chamber or circulating media from bipartite or tripartite bioengineered vessels. After 24 h and extensive washes, fluorescence was measured at 520 nm, excitation 490 nm. Points in graphed data represent individual bioengineered vessels, bars represent mean, error bars represent ±SEM and analysed by Student’s t-test or one way ANOVA *P < 0.05 and **P < 0.01. L: lumen

Bioengineered vessels similar to cortical penetrating arteries were fabricated using primary human EC, SMC and astrocytes (Fig. 6a). We previously reported that vessels lacking astrocytes (bipartite tissues) accumulate more Aβ42 than tissue with astrocytes (tripartite tissues) [31], which we confirmed here (Fig. 6b). We previously hypothesised that this difference might be due to increased apoE levels in tissues with astrocytes [31]. Here we tested whether apoE secreted by astrocytes might also reduce Aβ42 binding to collagen-I. After tissue homogenization, fractionation by SDS-PAGE and immunoblotting, we first confirmed that astrocyte-secreted apoE was increased by 1.5-fold in the tissue and 2-fold in the antelumen chamber but not in the circulation media as measured by ELISA (Fig. 6c-d), confirming that native astrocyte-secreted apoE does not cross the EC barrier in bioengineered vessels. We then tested whether conditioned media extracted from the antelumen chamber or circulation of bi- and tripartite tissues reduces Aβ42 binding to collagen-I. We found that tripartite antelumen chamber media significantly reduced Aβ42 binding to collagen-I compared to bipartite chamber media whereas no differences were observed with circulation media. Finally, we found no differences when comparing Aβ42 binding to the protein vehicle control BSA (Fig. 6e). Together, these data suggest that apoE, and/or other factors, secreted by astrocytes prevents binding of Aβ to vascular collagen-I, thereby reducing CAA in bioengineered vessels.

## Discussion

ApoE is the major genetic risk factor for late onset AD and plays multiple roles in AD pathogenesis including brain lipid transport, regulation of Aβ aggregation and clearance, neuronal function and signalling, cerebrovascular integrity, glucose metabolism, neuroinflammation, and mitochondrial function [34]. For decades, apoE has been presumed to affect AD through its production and actions within the brain, as apoE is the major apolipoprotein secreted by astrocytes, microglia, pericytes and stressed neurons [34]. Although apoE is also produced by the liver and peripheral tissue macrophages, findings from liver transplant patients showed that apoE does not cross the blood-brain barrier and, as such, the brain and plasma pools of apoE are distinct [54]. Potential roles for peripheral apoE in AD have therefore been understudied. Here we show that, as part of circulating HDL complexes, peripheral apoE can significantly reduce CAA in bioengineered vessels, raising the hypothesis that apoE-enriched HDL particles may promote vascular resilience to Aβ. Our results support new perspectives on apoE’s role in AD pathogenesis, including: 1) that circulating apoE may play a greater role in Aβ clearance than previously appreciated, and 2) that interventions based on apoE-enriched HDL may be effective in promoting Aβ clearance across the vascular wall, raising the possibility of a systemic preventative, companion or therapeutic approach for AD.

This study defines multiple mechanisms by which HDL promotes cerebrovascular health in the context of AD, with a focus on the capacity of HDL to reduce Aβ accumulation in the vascular wall. We first demonstrated that HDL acts independently of SR-BI to decrease vascular Aβ accumulation, which is in contrast to the SR-BI dependent mechanism by which HDL reduces Aβ-induced inflammation [32], representing a major bifurcation in mechanism along the anti-inflammatory and anti-CAA effects of HDL. These data are aligned with previous studies reporting increased perivascular macrophages in the brains of SR-BI^+/−^ and SR-BI ^−/−^ mice [55]. Additional supporting evidence for the role of SR-BI includes enhanced Aβ deposition in the brain and cerebrovasculature due to defective Aβ clearance by perivascular macrophages in J20 AD mice on an SR-BI ^+/−^ background [55]. Here we used human bioengineered vessels to show that blocking SR-BI does not diminish the ability of HDL to reduce vascular Aβ42 accumulation in the absence of circulating monocytes and perivascular macrophages, suggesting that SR-BI may be a key player in the inflammatory response to Aβ in the vascular wall. Further work will be needed to specifically understand the role of transmigrating monocytes and perivascular macrophages in CAA and Aβ clearance in both human and animal model systems, to further refine our understanding of the roles of HDL and SR-BI in this process.

With respect to CAA, our findings support four pathways by which HDL contributes to reduced vascular Aβ deposition, namely i) reducing Aβ binding to collagen-I, ii) reducing collagen-I production from SMC, and iii) forming a complex that maintains Aβ in a soluble state, and iv) reducing Aβ uptake into SMC that may be associated with reduced LRP1 protein levels or other to-be-defined mechanisms. Importantly, some of these processes depend on the apoE content of HDL particles. As a first step, we found that HDL needs to enter the vascular wall to affect SMC and interact with the ECM, however, exactly how HDL and HDL-associated lipoproteins enter the cerebrovascular wall remains to be elucidated. Once in the vascular wall, one mechanism by which HDL may attenuate Aβ deposition in the vasculature is by reducing LRP1-mediated Aβ uptake into SMC. We showed that HDL modestly reduces LRP1 levels in SMC monolayer cultures yet we did not observe this phenomenon in bioengineered vessels. We hypothesize that differences in culture times and culture complexity might underlie this discrepancy. Specifically, SMC are cultured alone in 2D culture for days whereas in bioengineered vessels, SMC are co-cultured with EC for several weeks and thus have an extended time to produce vascular ECM [37]. A subtle decrease in LRP1 level as observed in 2D might easily be lost in the increased complexity of 3D culture. Still, blocking LRP1 using RAP decreased the accumulation of RIPA-soluble Aβ42 in engineered vessels, suggesting that preventing Aβ uptake into SMC may promote its acute clearance through the vessel wall.

However, this pathway may be a minor mechanism, as only a small proportion of Aβ42 was taken up by SMC whereas the vast majority is bound to the ECM produced by SMC. Using co-localization studies in human AD cortex, we showed that vascular Aβ was primarily located outside of the cerebrovascular cells and associated with ECM matrix, in particular collagen-I. We did not observe specific vesicular staining in the vasculature of human cortical samples that would suggest uptake by SMC. We previously reported that HDL did not alter LRP1 protein levels in EC [32]. Although a previous study reported that blocking LRP1-mediated Aβ42 uptake by SMC reduces cell death and decreases CAA risk [56], another study reported that LRP1^−/−^ mice have increased vascular Aβ accumulation [48]. Furthermore, Bell and colleagues elegantly showed that overexpression of serum response factor and myocardin in SMC reduces LRP1 expression, induces arterial hypercontractility, and reduces cerebral blood flow (CBF), ultimately leading to increased CAA in mice [57]. The discrepancy might be explained by differences in experimental design, as we investigated acute responses in our human bioengineered vessel compared to monitoring outcomes for several months for the murine studies. We also focused on only one Aβ species in our study. Further studies on the role of LRP1, specially using cell-type specific LRP1^−/−^ models, will be needed to understand both the short- and long-term roles of LRP1 on the uptake of different species of Aβ by SMC and EC in the vasculature.

With respect to HDL’s effect on ECM, our results demonstrate that HDL significantly reduces collagen-I levels produced by SMC in 2D monoculture. We tested these effects in the presence and absence of Aβ42 and confirm that, in both cases, collagen-I levels were significantly reduced, suggesting that HDL’s effect on collagen expression operates independently of Aβ42. Our observations are consistent with previous studies demonstrating that apoE-containing HDL decreases ECM gene expression in aortic SMC [52]. Here also, we did not observe a significant difference in collagen-I level in bioengineered vessels after HDL treatment. Again, we hypothesize that discrepancy between 2D monoculture and 3D bioengineered vessels might be explained by the different cultured conditions, as experiments in 2D culture were performed 24 h after seeding SMC when the cells are in a growth phase and start to secret collagen-I, whereas experiments utilizing 3D bioengineered vessels begin after SMC have been maintained in culture for at least a month. Previous studies have demonstrated that collagen-I deposits within the vascular wall of bioengineered vessels over time [37, 58]. Because we measured collagen-I level only 24 h after HDL treatment, it is possible that collagen-I levels are either more susceptible to be altered by HDL in 2D culture, or that HDL-modification of collagen-I levels in 3D bioengineered vessels requires more than 24 h to yield significant effects due to the relatively greater amount of deposited collagen-I in the 3D vs. 2D models. As HDL reduces CAA in bioengineered vessels within 24 h of treatment, we hypothesize that reduced collagen-I levels is unlikely to play a major acute role in reducing CAA, although lower collagen deposition over the long term could preserve vascular reactivity and thus maintain vascular and brain health. This is important as the cerebrovasculature stiffens during aging due to altered ECM composition with increased collagen and reduced elastin levels. Moreover, mild cognitively impaired and AD individuals have reduced cerebrovascular reactivity [59], indicative of increased vessel stiffness.

Our results demonstrate that HDL treatment alters collagen-I size distribution, which associates with the apoE and apoA-II moieties in HDL particles. Further, Aβ42 accumulation, collagen-I size distribution and Aβ42 binding to collagen-I are all dependent on the presence of apoE on HDL particles. These results are highly relevant as they support emerging hypotheses highlighting the role of peripheral apoE as an important contributor to the onset and progression of AD [60–62]. ApoE-containing HDL only represents 6 to 9% of total HDL particles in the plasma of normolipidemic individuals with the remaining apoE being associated with larger lipoproteins such as VLDL and LDL [33]. Here we show that a mixture of LDL, ILDL and VLDL fails to prevent Aβ42 accumulation in engineered vessels, supporting the specificity of HDL particles in this process. Further work will be required to understand how apoE-containing HDL particles specifically reduce CAA and if apoE isoforms differentially affect this process. Notably, mice expressing human apoE4 have disturbed perivascular drainage and increased vascular basement membrane collagen-IV levels at 3 months of age but decreased levels at 16 months of age compared to apoE3 mice [63]. *APOE4/4* is also associated with a thinning of microvascular basement membrane [64] and altered ECM composition with deposition of fibrin(ogen) compared to *APOE3/3* [65]*.* Importantly, whether brain-derived or peripheral apoE drives these vascular changes is not yet known.

As Aβ42 is a hydrophobic peptide and HDL carries lipids, we also tested whether HDL and Aβ42 can form a physical complex. Our gel-filtration chromatography experiments support this possibility, as elution fractions differed when Aβ42 was incubated with HDL compared to BSA as a protein control, suggesting specific binding of Aβ42 to HDL. Additionally, HDL complexed with Aβ exhibited lower cholesterol efflux activity, suggesting that Aβ binding to HDL may compromise one of HDL’s most well-described functions. Although these findings suggest that HDL can form a complex with Aβ42, further work will be needed to establish the exact interaction mechanisms. Nevertheless, the formation of a potential HDL-Aβ complex suggests that HDL could compete with and prevent Aβ42 binding to collagen-I. In line with this finding, several groups report an interaction of HDL and Aβ, with HDL being the major binding partner of Aβ fibrils in plasma [22, 66].

Finally, apoE-containing HDL has recently been established as a biomarker for coronary artery disease (CAD) risk [67]. As assays to selectively measure the proportion of HDL particles containing apoE become more readily available, it will be important to determine whether the levels of circulating apoE-containing HDL help to predict the risk of CAA, total brain Aβ accumulation, and cognitive decline in AD patients.

Our study has several limitations. One is that we used readily obtainable umbilical cords to isolate the EC and SMC used to fabricate bioengineered vessels, as primary cerebrovascular EC have a slower growth rate and commercial supplies are limited compared to the demands of the bioengineered vascular model, and induced pluripotent stem cell (iPSC) EC have greater batch-to-batch variation during differentiation. Although cord-derived cells are not brain-like, several studies demonstrate that cord cells are plastic and can acquire specific organ features depending on the microenvironment in which they are cultured. In particular, several studies showed that HUVEC can become reprogrammed to express selective BBB marker proteins when cultured in the brain environment [31, 68, 69]. Furthermore, we previously showed that the histological structure as well as Aβ accumulation and transport were similar between vessels bioengineered using primary human brain vascular cells vs. umbilical cord cells [31], raising confidence on the validity of our findings. A second limitation concerns the number of SMC layers, which is greater in bioengineered vessels compared to the few layers of SMC in human cerebral arteries [70]. While this difference might influence our results, it is important to note that both in post-mortem human tissues and in bioengineered vessels the vast majority of Aβ co-localizes with ECM and not within SMC. A third limitation is that, similar to previous in vitro studies [71, 72], the Aβ42 concentrations used here are supraphysiological relative to the levels in human brain, CSF or cerebral interstitial fluid, which was required to be within the detection limits of our ELISA assay [73–75]. Future studies using more sensitive Aβ assays that also use native neuronally-produced Aβ in bioengineered tissues would circumvent this limitation. Finally, the concentration of HDL used here is lower than in to normolipidomic individuals. HDL concentrations within plasma which roughly corresponds to 140 mg apoA-I /dL [76]. We circulated 200 μg protein/mL HDL in our experiments, similar to what has previously been published for other in vitro studies [37, 77], as we are limited by the availability of HDL, in particular HDL enriched in apoE.

## Conclusions

**Fig. 7:**
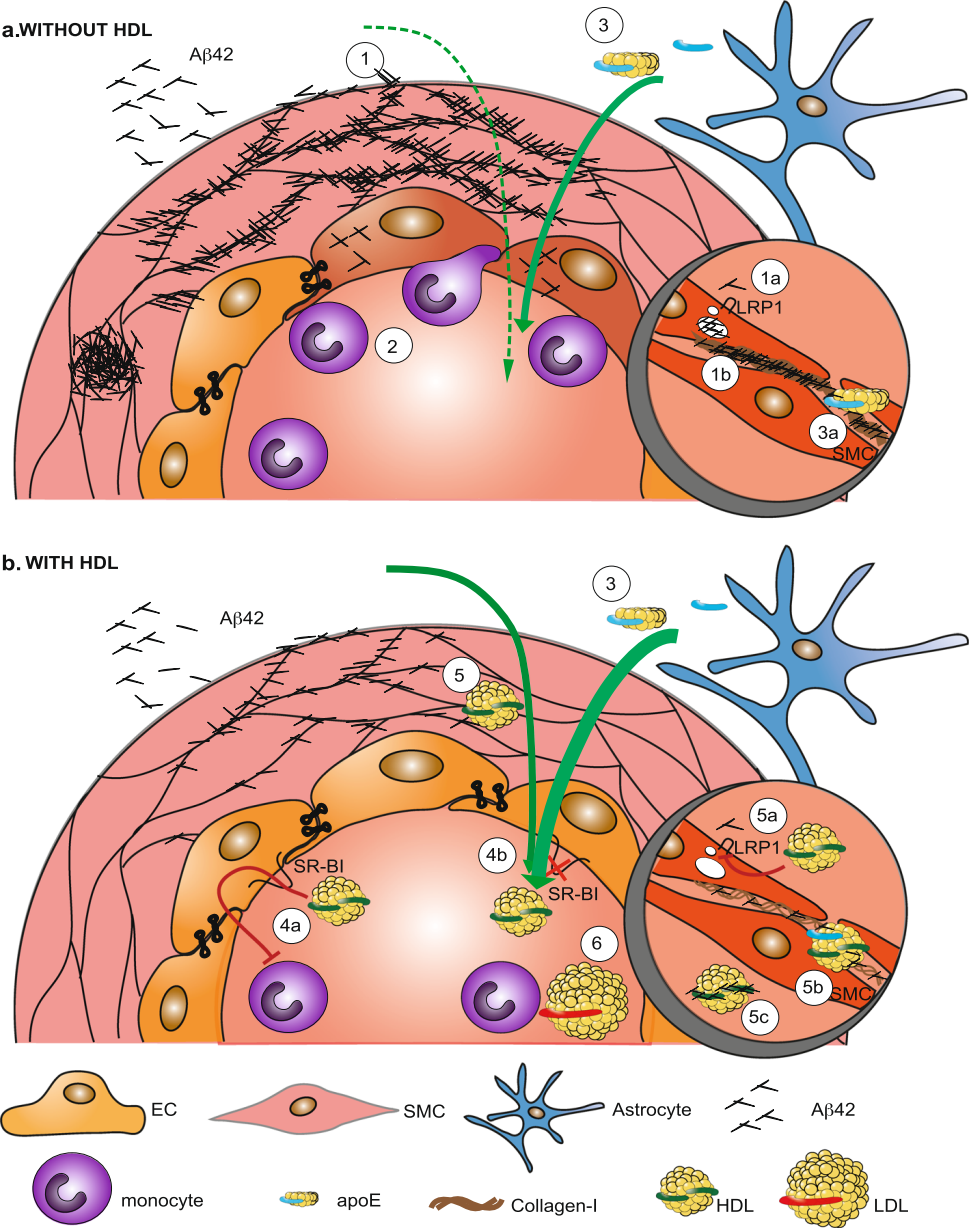
Schematic of the mechanisms by which HDL is hypothesized to protect cerebrovascular functions in AD. **a** In the absence of HDL, when Aβ peptides enter the vessel wall they are either taken up by SMC via LRP1 (**1a**) or become trapped in ECM, particularly when enriched in collagen-I (**1b**). **2**) Accumulation of Aβ in the vessel wall induces endothelial activation and subsequent binding and transmigration of blood-derived circulating monocytes. **3**) Astrocytes on the antelumen of penetrating arteries secret apoE that reduces collagen-I levels in the vascular wall. **b** in the presence of HDL, **4**) HDL reduces monocyte binding via SR-BI (**4a**), whereas reduction of CAA is independent of SR-BI (**4b**). **5**) HDL enters the cerebrovascular wall through undefined mechanisms and reduce Aβ uptake by SMC maybe by reducing LRP1 levels (**5a**), bind and remodel collagen-I fibres via HDL-apoE particles (**5b**) and form a complex with Aβ (**5c**), all of which increase luminal recovery of Aβ. **6**) The reduction of CAA is specific to HDL

In conclusion, we define multiple pathways by which HDL exhibits beneficial properties relevant to AD. HDL uses SR-BI to attenuate Aβ-mediated endothelial inflammation and employs multiple SR-BI-independent pathways to attenuate Aβ deposition in engineered vessels. HDL reduces Aβ42 uptake by SMC perhaps by reducing LRP1 levels, alters binding of Aβ42 to collagen-I likely by forming an HDL-Aβ complex, and diminishes collagen-I production by SMC (Fig. 7). Intriguingly, these are influenced by the presence of apoE on HDL particles. All of these mechanisms promote Aβ clearance through the vasculature, thereby reducing CAA. The findings in this study begin to elucidate an important mechanistic pathway concerning AD pathogenesis, identifies new potential therapeutic targets, and sheds new light on the role of peripherally-acting apoE in AD.

## Supplementary information

**Additional file 1: Supplemental Figure 1.** Schematic view of the bioengineered vessel and bioreactor. The left panel represents a schematic of the bioreactor design with the peristaltic pump, media container, circulation loop, and bioengineered vessel chamber. The bioengineered vessel separates the tissue chamber (brown) where Aβ is injected from the circulation loop (red) where HDL is circulated. The right panel depict the schematic of bioengineered vessels composed of EC (yellow), SMC (orange) without (bipartite) or with (tripartite) astrocyte (blue). Non-woven PGA/PCL scaffold is showed in white and defines the number of SMC layers. **Supplementary Figure 2.** HDL and mix LDL delay Aβ42 fibrillization. **(a**) Aβ fibrillization was measured in a Thioflavin T cell-free assay over 7200 min. (**b**) Maximal fluorescence, (**c**) time to half-maximal fluorescence (V50) and (**d**) lag phases were calculated using Boltzmann curve analysis (vehicle *R*^*2*^ = 0.91; HDL *R*^*2*^ = 0.94; LDL *R*^*2*^ = 0.4). Points in graphed data represent individual experiments, bars represent mean, error bars represent ±SEM and data are presented as mean +/− SEM and analysed by one way ANOVA *P < 0.05, **P < 0.01, ***P < 0.001 and ****P < 0.0001. **Supplementary Figure 3.** HDL does not reduce deposition of preformed Aβ42 oligomers or fibrils in engineered tissues. (**a**) 1 μM Aβ42 was injected into the tissue chamber concomitantly with circulation of 200 μg/mL HDL through the lumen before measuring Aβ42 deposition in the GluHCl fraction by ELISA and examining Thioflavin S staining (white) after 24 h (* remaining scaffold). (**b**) Aβ42 oligomers were prepared by incubating monomers in RPMI for 48 h at 4 °C before injection of 1 μM oligomers into the tissue chamber while circulating 200 μg/mL HDL through the lumen. After 24 h, Aβ was measured in both RIPA and GluHCl fractions by ELISA. (**c**) Aβ42 fibrils were prepared by incubating monomers in RPMI for 48 h at 37 °C before injection of 1 μM fibres into the tissue chamber while circulating 200 μg/mL HDL through the lumen. After 24 h, Aβ was measured in both RIPA and GluHCl fractions by ELISA. (**d**) 1 μM Aβ42 monomers were injected into the antelumen 48 h before circulating 200 μg/mL HDL through the lumen. After 24 h, Aβ42 deposition was measured in RIPA and GluHCl fractions by ELISA. Thioflavin-S staining are representative image of 3 individual tissues. Points in graphed data represent individual bioengineered vessels, bars represent mean, error bars represent ±SEM and analysed by Student’s t-test. **Supplementary Figure 4.** HDL reduces FITC-Aβ42 uptake by EC and HDL does not increase Aβ42 co-localization with lysosome in SMC. EC were grown in 2D monolayers for 2 d before treating with 1 μM FITC-Aβ42 without or with 200 μg/mL HDL for 3 h (**a**) or 24 h (**b**) before dissociating cells and counting using flow cytometry. (**c**) SMC were grown in chamber slides for 2 d before treating with 5 μM FITC-Aβ42 (depicted as yellow) without or with 200 μg/mL HDL. After 24 h, SMC were fixed and stained for the lysosomal marker LAMP1 (magenta) before imaging using confocal microscopy. Points in graphed data represent individual bioengineered vessels, bars represent mean, error bars represent ±SEM and analysed by Student’s t-test *P < 0.05 and **P < 0.01. Fluorescent images are representative of two separate experiments. **Supplementary Figure 5.** HDL does not alter Aβ42 entry from the circulation into the vascular wall. (**a**) 1 μM Aβ42 monomers were injecting into the tissue chamber with or without circulating 200 μg/mL of HDL through the lumen. After 24 h tissues were lysed in RIPA and RAGE levels were measured in bioengineered tissues using Western blot and normalized to GAPDH. 1 μM Aβ42 monomers were injecting either into the tissue chamber or the circulation loop with or without circulating 200 μg/mL of HDL through the lumen. After 24 h, Aβ42 was measured in the tissue chamber (**b**), circulation (**c**), and tissue lysed in RIPA (**d**) by ELISA. (**e**) 1 μM Aβ42 was injected into the tissue chamber with or without a blocking antibody against RAGE in the circulation. After 24 h, tissues were lysed in RIPA Aβ42 levels were measured by ELISA. Points in graphed data represent individual bioengineered vessels, bars represent mean, error bars represent ±SEM and analysed by Student’s t-test or one-way ANOVA *P < 0.05, **P < 0.01, ***P < 0.001 and ****P < 0.0001. **Supplementary Figure 6.** Aβ deposition co-localizes with vascular ECM in post mortem human cortex. Human cortex (Brodmann area 9) were stained for Aβ using 6E10 (yellow) and collagen-IV (**a**), laminin (**b**), biglycan (**c**), HSP2 (**d**) and versican (**e**) (magenta). Expanded views of a vessel with CAA (1) and cortical Aβ plaques (2) are shown on the right. Images are representative of four individual human donors. **Supplementary Figure 7.** Aβ42 forms a complex with HDL and reduces HDL’s cholesterol efflux activity. **(a)** 1 μM Aβ42 were incubated either with 200 μg/mL of HDL or BSA for 24 h at 37 °C before gel-filtration chromatography separation. HDL alone (**b**) or Aβ42 monomers alone (**c**) were separated by gel-filtration chromatography and dot blotted fractions were probed against Aβ (6E10) or HDL (apoA-II). Graphs and dot-blots show representative experiments from three individual FPLC runs. RAW 264.7 cells were loaded with H^3^-cholesterol and cholesterol efflux was measured in the presence of HDL (**d**), lipid-free apoA-I (**e**) or no acceptor (**f**), pre-incubated with or without 1 μM Aβ42 for 24 h. Points in graphed data represent individual experiments, bars represent mean, error bars represent ±SEM and analysed by Student’s t-test *P < 0.05, ***P < 0.001 and ****P < 0.0001.

## Data Availability

Raw data can be obtained from corresponding author.

## Abbreviations

AD: Alzheimer’s disease
Apo: apolipoprotein
Aβ: beta-amyloid
CAA: cerebral amyloid angiopathy
CAD: coronary artery diseases
EC: endothelial cells
ECM: extracellulat matrix
HDL: high-density lipoprotein
LDL: low-density lipoprotein
LRP1: low density lipoprotein receptor-related protein 1
RAGE: receptor for advanced glycation endproduct
RAP: receptor-associated protein
SMC: smooth-muscle cells
