# Supplementary material for: Cerebrovascular amyloid Angiopathy in bioengineered vessels is reduced by high-density lipoprotein particles enriched in Apolipoprotein E
Source: Mol Neurodegener. 2020 Mar 25;15:23. doi: 10.1186/s13024-020-00366-8 (PMC7093966; doi:10.1186/s13024-020-00366-8)
Supplement: Supplementary file 1 — Additional file 1: Supplemental Figure 1. Schematic view of the bioengineered vessel and bioreactor. The left panel represents a schematic of the bioreactor design with the peristaltic pump, media container, circulation loop, and bioengineered vessel chamber. The bioengineered vessel separates the tissue chamber (brown) where Aβ is injected from the circulation loop (red) where HDL is circulated. The right panel depict the schematic of bioengineered vessels composed of EC (yellow), SMC (orange) without (bipartite) or with (tripartite) astrocyte (blue). Non-woven PGA/PCL scaffold is showed in white and defines the number of SMC layers. Supplementary Figure 2. HDL and mix LDL delay Aβ42 fibrillization. (a) Aβ fibrillization was measured in a Thioflavin T cell-free assay over 7200 min. (b) Maximal fluorescence, (c) time to half-maximal fluorescence (V50) and (d) lag phases were calculated using Boltzmann curve analysis (vehicle R2 = 0.91; HDL R2 = 0.94; LDL R2 = 0.4). Points in graphed data represent individual experiments, bars represent mean, error bars represent ±SEM and data are presented as mean +/− SEM and analysed by one way ANOVA *P < 0.05, **P < 0.01, ***P < 0.001 and ****P < 0.0001. Supplementary Figure 3. HDL does not reduce deposition of preformed Aβ42 oligomers or fibrils in engineered tissues. (a) 1 μM Aβ42 was injected into the tissue chamber concomitantly with circulation of 200 μg/mL HDL through the lumen before measuring Aβ42 deposition in the GluHCl fraction by ELISA and examining Thioflavin S staining (white) after 24 h (* remaining scaffold). (b) Aβ42 oligomers were prepared by incubating monomers in RPMI for 48 h at 4 °C before injection of 1 μM oligomers into the tissue chamber while circulating 200 μg/mL HDL through the lumen. After 24 h, Aβ was measured in both RIPA and GluHCl fractions by ELISA. (c) Aβ42 fibrils were prepared by incubating monomers in RPMI for 48 h at 37 °C before injection of 1 μM fibres into the tissue chamber while circulat [file 13024_2020_366_MOESM1_ESM.pdf]

Sup. Fig. 1

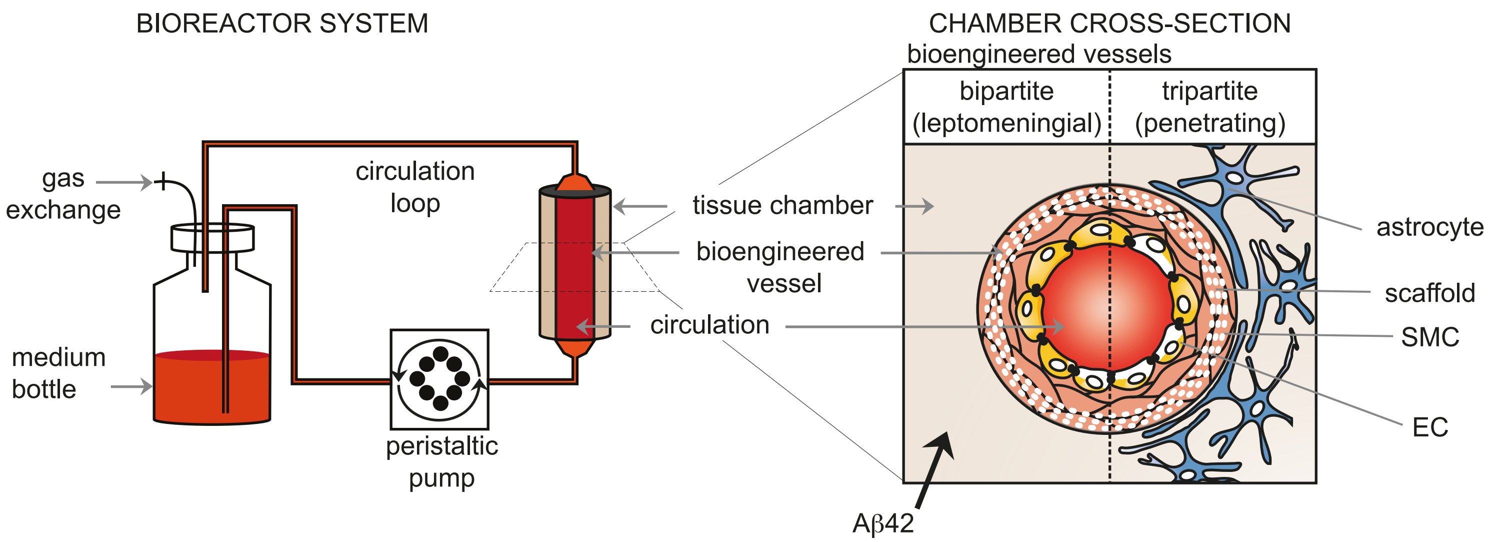
BIOREACTOR SYSTEM
gas exchange
circulation loop
medium bottle
peristaltic pump
tissue chamber
bioengineered vessel
circulation
CHAMBER CROSS-SECTION
bioengineered vessels
bipartite (leptomeningial)
tripartite (penetrating)
astrocyte
scaffold
SMC
EC
Aβ42

Supplemental Figure 1. Schematic view of the bioengineered vessel and bioreactor.

The left panel represents a schematic of the bioreactor design with the peristaltic pump, media container, circulation loop, and bioengineered vessel chamber. The bioengineered vessel separates the tissue chamber (brown) where Aβ is injected from the circulation loop (red) where HDL is circulated. The right panel depict the schematic of bioengineered vessels composed of EC (yellow), SMC (orange) without (bipartite) or with (tripartite) astrocyte (blue). Non-woven PGA/PCL scaffold is showed in white and defines the number of SMC layers.

Sup Figure 2

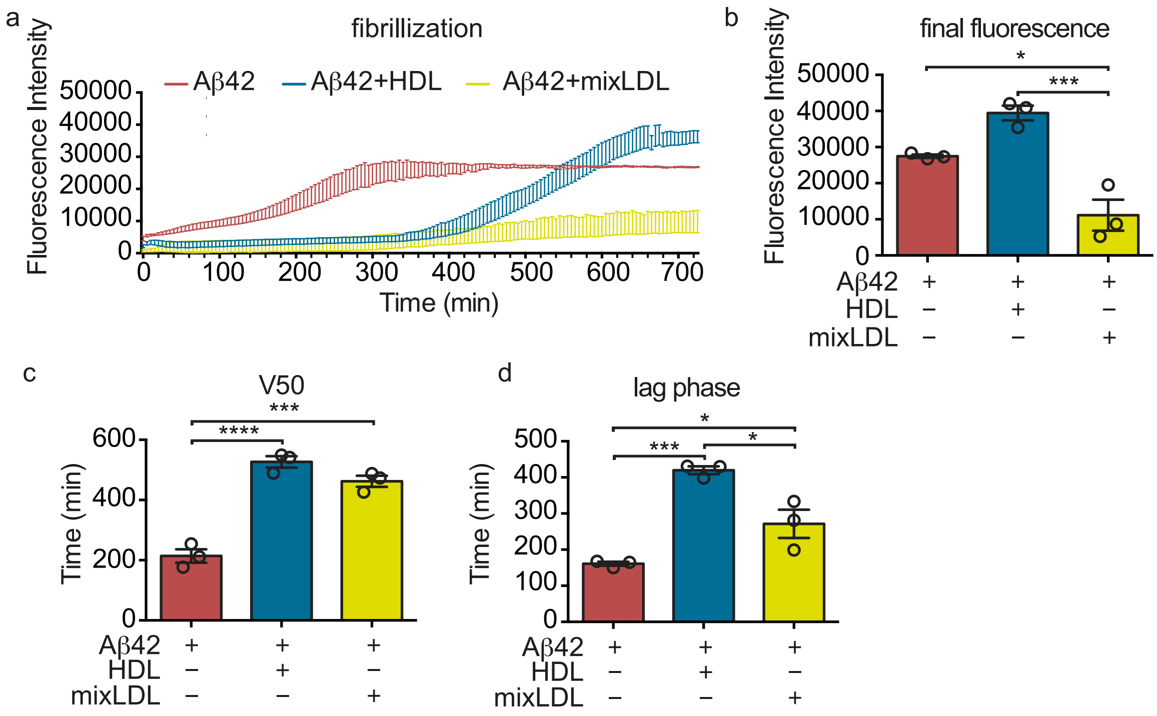

a
fibrillization
Aβ42
Aβ42+HDL
Aβ42+mixLDL
Fluorescence Intensity
50000
40000
30000
20000
10000
0
0
100
200
300
400
500
600
700
Time (min)
b
final fluorescence
Fluorescence Intensity
*
***
Aβ42 + + +
HDL − + −
mixLDL − − +
c
V50
Time (min)
****
***
600
400
200
0
Aβ42 + + +
HDL − + −
mixLDL − − +
d
lag phase
Time (min)
*
***
*
500
400
300
200
100
0
Aβ42 + + +
HDL − + −
mixLDL − − +

Supplementary Figure 2. HDL and mix LDL delay Aβ42 fibrillization.

(**a**) Aβ fibrillization was measured in a Thioflavin T cell-free assay over 7200 minutes. (**b**) Maximal fluorescence, (**c**) time to half-maximal fluorescence (V50) and (**d**) lag phases were calculated using Boltzmann curve analysis (vehicle R^2=0.91; HDL R^2=0.94; LDL R^2=0.4). Points in graphed data represent individual experiments, bars represent mean, error bars represent ±SEM and data are presented as mean +/- SEM and analysed by one way ANOVA *P<0.05, **P<0.01, ***P<0.001 and ****P<0.0001.

Sup Figure 3

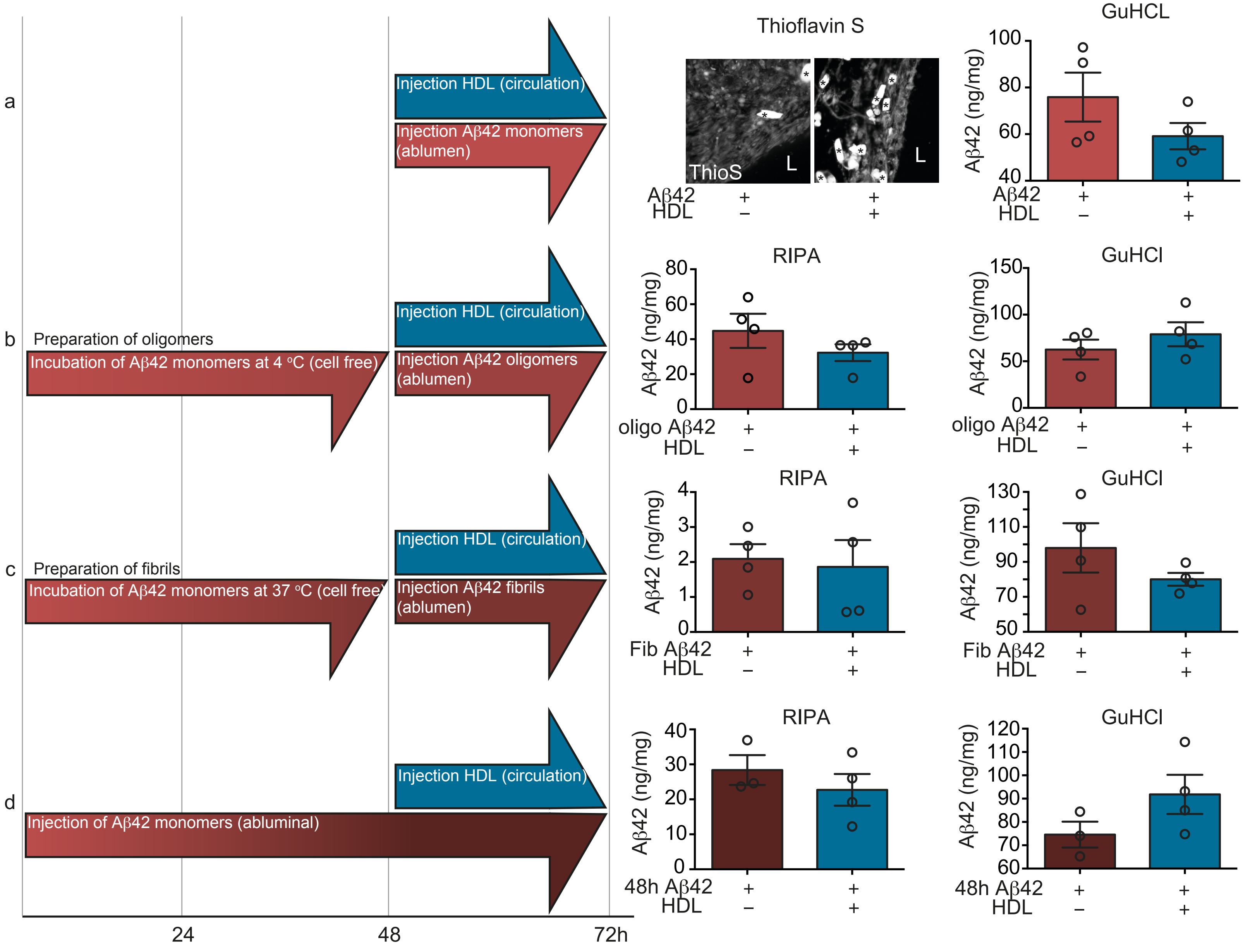

a
b
c
d
Injection HDL (circulation)
Injection Aβ42 monomers (ablumen)
Preparation of oligomers
Incubation of Aβ42 monomers at 4 °C (cell free)
Injection Aβ42 oligomers (ablumen)
Preparation of fibrils
Incubation of Aβ42 monomers at 37 °C (cell free)
Injection Aβ42 fibrils (ablumen)
Injection of Aβ42 monomers (abluminal)
24
48
72h
Thioflavin S
ThioS
L
Aβ42 + +
HDL – +
GuHCL
RIPA
GuHCl
Aβ42 (ng/mg)
oligo Aβ42 + +
Fib Aβ42 + +
48h Aβ42 + +

Supplementary Figure 3. HDL does not reduce deposition of preformed Aβ42 oligomers or fibrils in engineered tissues.

(**a**) 1 μM Aβ42 was injected into the tissue chamber concomitantly with circulation of 200 μg/mL HDL through the lumen before measuring Aβ42 deposition in the GluHCl fraction by ELISA and examining Thioflavin S staining (white) after 24 h (* remaining scaffold). (**b**) Aβ42 oligomers were prepared by incubating monomers in RPMI for 48 h at 4 ^{o}C before injection of 1 μM oligomers into the tissue chamber while circulating 200 μg/mL HDL through the lumen. After 24 h, Aβ was measured in both RIPA and GluHCl fractions by ELISA. (**c**) Aβ42 fibrils were prepared by incubating monomers in RPMI for 48 h at 37 ^{o}C before injection of 1 μM fibres into the tissue chamber while circulating 200 μg/mL HDL through the lumen. After 24 h, Aβ was measured in both RIPA and GluHCl fractions by ELISA. (**d**) 1 μM Aβ42 monomers were injected into the antelumen 48 h before circulating 200 μg/mL HDL through the lumen. After 24 h, Aβ42 deposition was measured in RIPA and GluHCl fractions by ELISA. Thioflavin-S staining are representative image of 3 individual tissues. Points in graphed data represent individual bioengineered vessels, bars represent mean, error bars represent ±SEM and analysed by Student's t-test.

Sup Figure 4

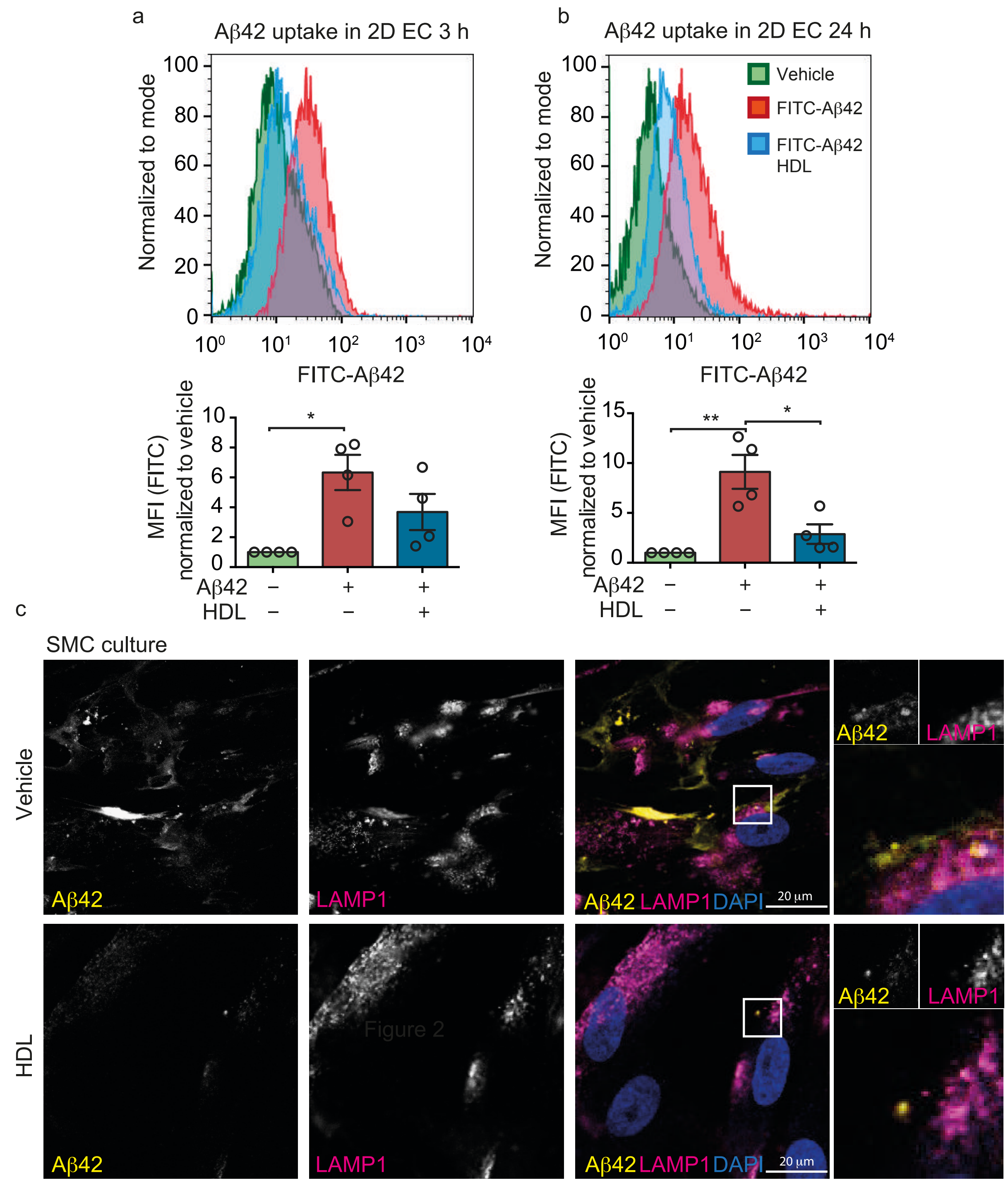
a
Aβ42 uptake in 2D EC 3 h
Normalized to mode
FITC-Aβ42
MFI (FITC) normalized to vehicle
Aβ42
HDL
b
Aβ42 uptake in 2D EC 24 h
Vehicle
FITC-Aβ42
FITC-Aβ42 HDL
c
SMC culture
Vehicle
HDL
Aβ42
LAMP1
DAPI
20 µm

Supplementary Figure 4. HDL reduces FITC-Aβ42 uptake by EC and HDL does not increase Aβ42 co-localization with lysosome in SMC.

EC were grown in 2D monolayers for 2 d before treating with 1 μM FITC-Aβ42 without or with 200 μg/mL HDL for 3 h (**a**) or 24 h (**b**) before dissociating cells and counting using flow cytometry. (**c**) SMC were grown in chamber slides for 2 d before treating with 5 μM FITC-Aβ42 (depicted as yellow) without or with 200 μg/mL HDL. After 24 h, SMC were fixed and stained for the lysosomal marker LAMP1 (magenta) before imaging using confocal microscopy. Points in graphed data represent individual bioengineered vessels, bars represent mean, error bars represent ±SEM and analysed by Student's t-test $^{*}P<0.05$ and $^{**}P<0.01$. Fluorescent images are representative of two separate experiments.

Sup fig 5

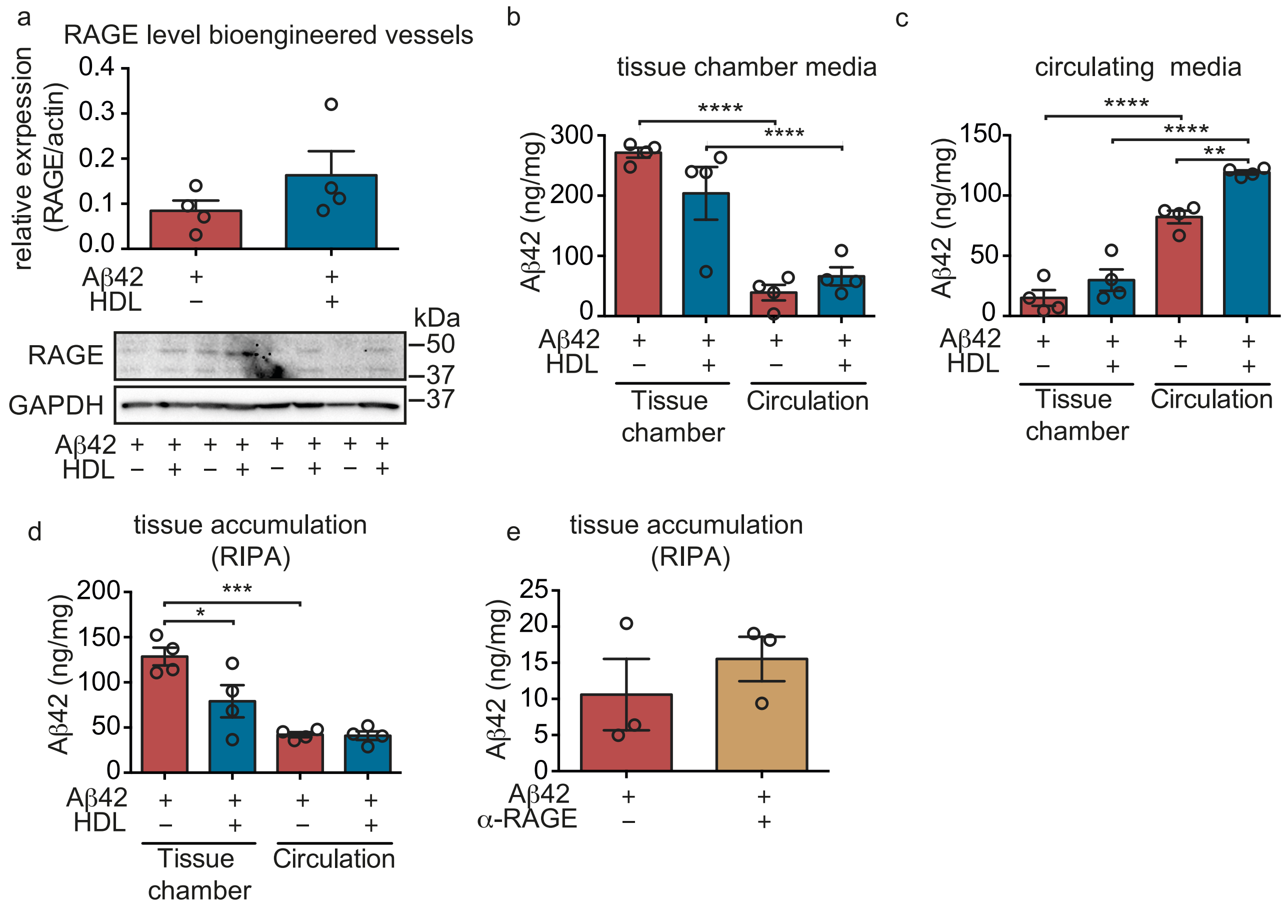
a
RAGE level bioengineered vessels
relative exrpession (RAGE/actin)
0.4
0.3
0.2
0.1
0.0
Aβ42 + +
HDL – +
kDa
RAGE
50
37
GAPDH
37
Aβ42 + + + + + + + +
HDL – + – + – + – +
b
tissue chamber media
Aβ42 (ng/mg)
300
200
100
0
****
****
Aβ42 + + + +
HDL – + – +
Tissue chamber
Circulation
c
circulating media
Aβ42 (ng/mg)
150
100
50
0
****
****
**
Aβ42 + + + +
HDL – + – +
Tissue chamber
Circulation
d
tissue accumulation (RIPA)
Aβ42 (ng/mg)
200
150
100
50
0
***
*
Aβ42 + + + +
HDL – + – +
Tissue chamber
Circulation
e
tissue accumulation (RIPA)
Aβ42 (ng/mg)
25
20
15
10
5
0
Aβ42 + +
α-RAGE – +

Supplementary Figure 5. HDL does not alter Aβ42 entry from the circulation into the vascular wall.

(**a**) 1 μM Aβ42 monomers were injecting into the tissue chamber with or without circulating 200 μg/mL of HDL through the lumen. After 24 h tissues were lysed in RIPA and RAGE levels were measured in bioengineered tissues using Western blot and normalized to GAPDH. 1 μM Aβ42 monomers were injecting either into the tissue chamber or the circulation loop with or without circulating 200 μg/mL of HDL through the lumen. After 24 h, Aβ42 was measured in the tissue chamber (**b**), circulation (**c**), and tissue lysed in RIPA (**d**) by ELISA. (**e**) 1 μM Aβ42 was injected into the tissue chamber with or without a blocking antibody against RAGE in the circulation. After 24 h, tissues were lysed in RIPA Aβ42 levels were measured by ELISA. Points in graphed data represent individual bioengineered vessels, bars represent mean, error bars represent ±SEM and analysed by Student's t-test or one-way ANOVA *P<0.05, **P<0.01, ***P<0.001 and ****P<0.0001.

Sup Fig 6

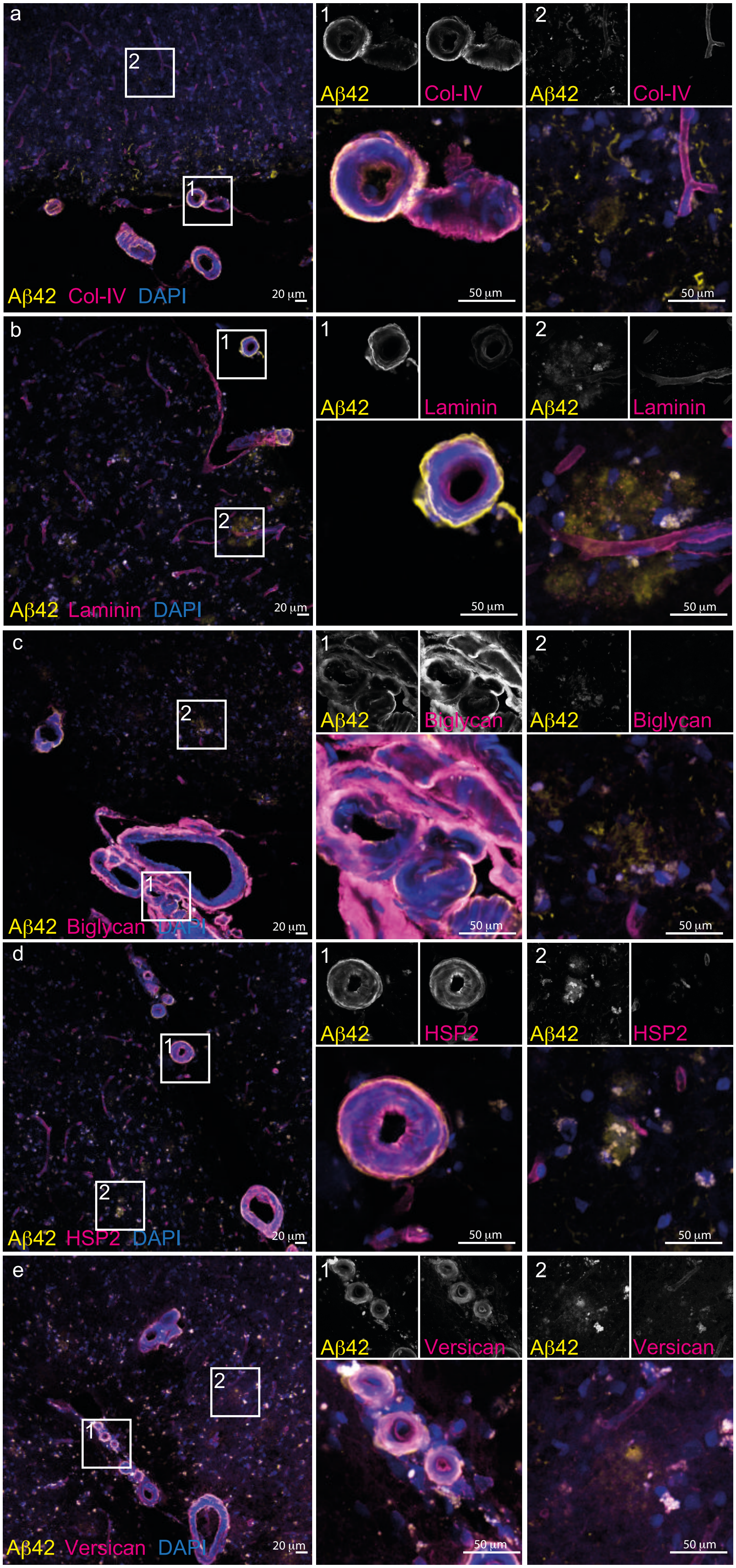
a
b
c
d
e
Aβ42 Col-IV DAPI
Aβ42 Laminin DAPI
Aβ42 Biglycan DAPI
Aβ42 HSP2 DAPI
Aβ42 Versican DAPI
Aβ42
Col-IV
Laminin
Biglycan
HSP2
Versican
1
2
20 µm
50 µm

Supplementary Figure 6. Aβ deposition co-localizes with vascular ECM in *post mortem* human cortex.

Human cortex (Brodmann area 9) were stained for Aβ using 6E10 (yellow) and collagen-IV (**a**), laminin (**b**), biglycan (**c**), HSP2 (**d**) and versican (**e**) (magenta). Expanded views of a vessel with CAA (1) and cortical Aβ plaques (2) are shown on the right. Images are representative of four individual human donors.

Sup fig 7

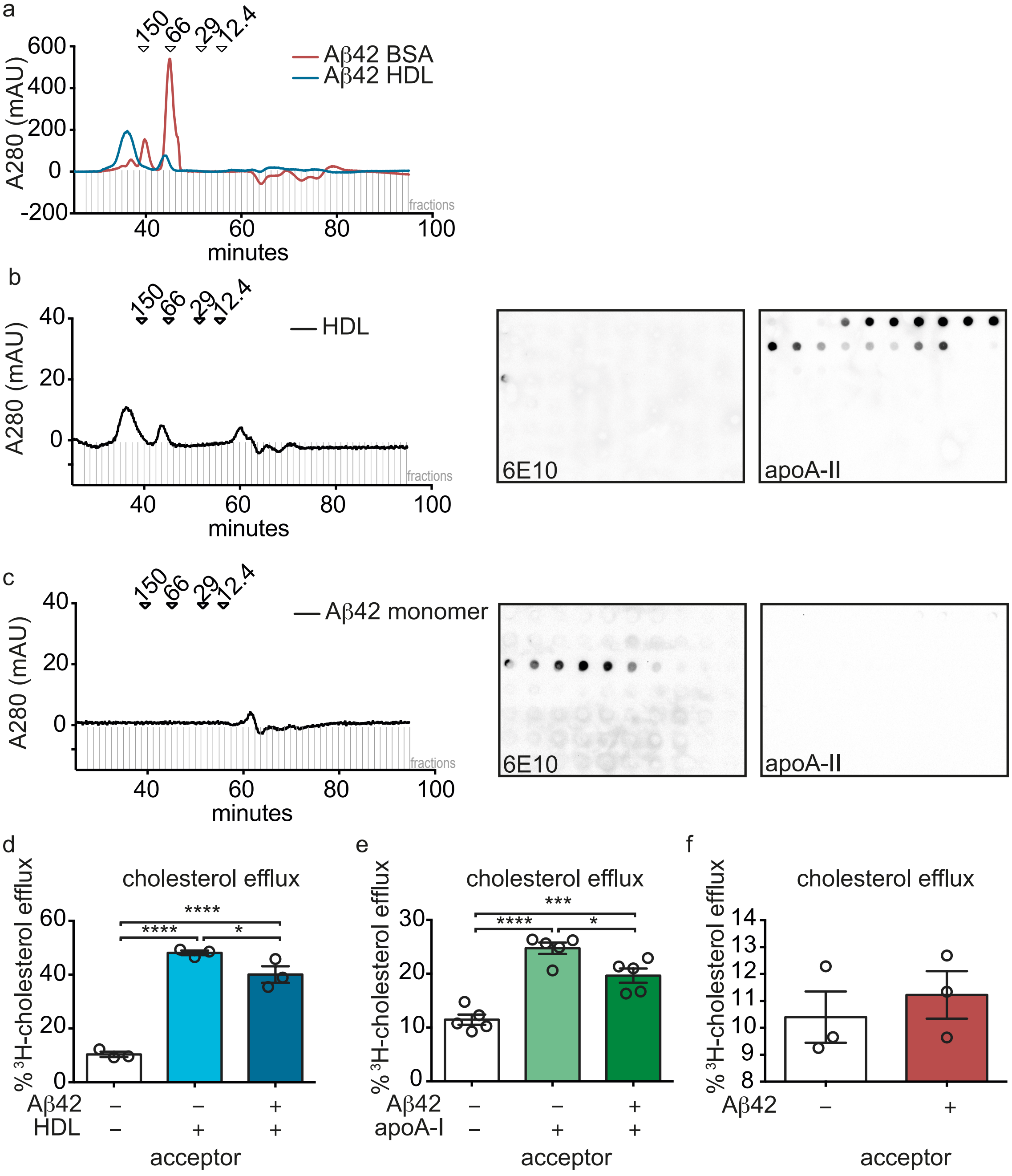

a
150
66
29
12.4
Aβ42 BSA
Aβ42 HDL
A280 (mAU)
600
400
200
0
-200
fractions
40
60
80
100
minutes
b
HDL
40
20
0
6E10
apoA-II
c
Aβ42 monomer
d
cholesterol efflux
% ³H-cholesterol efflux
****
*
Aβ42
HDL
acceptor
e
***
apoA-I
f

Supplementary Figure 7. Aβ42 forms a complex with HDL and reduces HDL's cholesterol efflux activity.

(a) 1 μM Aβ42 were incubated either with 200 μg/mL of HDL or BSA for 24 h at 37 ^{o}C before gel-filtration chromatography separation. HDL alone (**b**) or Aβ42 monomers alone (**c**) were separated by gel-filtration chromatography and dot blotted fractions were probed against Aβ (6E10) or HDL (apoA-II). Graphs and dot-blots show representative experiments from three individual FPLC runs. RAW 264.7 cells were loaded with H^3-cholesterol and cholesterol efflux was measured in the presence of HDL (**d**), lipid-free apoA-I (**e**) or no acceptor (**f**), pre-incubated with or without 1 μM Aβ42 for 24 h. Points in graphed data represent individual experiments, bars represent mean, error bars represent ±SEM and analysed by Student's t-test *$P<0.05$, ***$P<0.001$ and ****$P<0.0001$.
